# Cell line-specific gene network enrichment analysis for interpreting continuous phenotypes

**DOI:** 10.1093/bib/bbag413

**Published:** 2026-07-30

**Authors:** Heewon Park, Seiya Imoto, Satoru Miyano

**Affiliations:** School of Mathematics, Statistics and Data Science, Sungshin Women’s University, 2, 34 dagil, Bomun-ro, Seongbuk-gu, Seoul, 02844, Republic of Korea; M&D Data Science Center, Institute of Integrated Research, Institute of Science Tokyo, 1-5-45 Yushima, Bunkyo-ku, Tokyo 113-8510, Japan; Human Genome Center, Institute of Medical Science, University of Tokyo, 4-6-1 Shirokanedai, Minato-ku, Tokyo, 108-8639, Japan; Human Genome Center, Institute of Medical Science, University of Tokyo, 4-6-1 Shirokanedai, Minato-ku, Tokyo, 108-8639, Japan; M&D Data Science Center, Institute of Integrated Research, Institute of Science Tokyo, 1-5-45 Yushima, Bunkyo-ku, Tokyo 113-8510, Japan; Human Genome Center, Institute of Medical Science, University of Tokyo, 4-6-1 Shirokanedai, Minato-ku, Tokyo, 108-8639, Japan

**Keywords:** enrichment analysis, cell line-specific gene network, continuous phenotypes, leukemia

## Abstract

Gene network enrichment analysis (GNEA) offers a robust approach for interpreting the complex molecular mechanisms underlying phenotypic variability. Despite its utility, prevailing GNEA methodologies are predominantly optimized for binary phenotypes, leading to substantial information loss when applied to continuous biological traits such as drug sensitivity, cancer progression, etc. Additionally, traditional enrichment strategies often exhibit conceptual inconsistency between their null models and test hypotheses, as they rely on permuting phenotype labels rather than genes. To address these limitations, we introduce cell line-specific gene network enrichment analysis (CellGNEA), a computational strategy designed to identify pathway-level molecular interactions associated with continuous phenotypes in a cell line-specific manner. CellGNEA constructs gene regulatory networks tailored to individual cell lines and assesses molecular interplays within these networks by integrating multiple network-derived metrics, including clustering coefficient, PageRank, and regulatory effects. Associations between gene networks and continuous phenotypes are quantified using a Kolmogorov–Smirnov-based statistic, with statistical significance determined via a gene permutation strategy that aligns the null model with the tested hypothesis. Monte Carlo simulation studies indicate that CellGNEA exhibits robustness and enhanced sensitivity in detecting network enrichment linked to continuous phenotypes. Applications of CellGNEA to drug sensitivity-specific gene networks enable the identification of leukemia-related pathways with molecular interactions significantly associated with therapeutic response. Notably, our analysis revealed that imatinib, quizartinib, and ruxolitinib consistently correlate with network-level remodeling across acute myeloid leukemia, myelodysplastic syndrome, and chronic myeloid leukemia pathways, and identified resistance-associated genes including PTPN11, MS4A1, and BTK. Overall, CellGNEA establishes a systematic, scalable framework for functional network analysis of continuous phenotypes, facilitating comprehensive characterization of cell line-specific biological properties and providing valuable insights for systems biology and precision medicine.

## Introduction

High-throughput genomic technologies have facilitated systematic exploration of the molecular mechanisms underlying complex diseases and phenotypic variability. With advancements in these technologies, gene network analysis has garnered increasing attention as an effective framework; heterogeneous gene networks provide robust models for capturing coordinated gene interactions and clarifying system-level disruptions associated with diverse disease processes. While multiple computational approaches, such as Bayesian gene network [1], $L_{1}$-type regularization [2], and graphical LASSO [3], have been developed to infer gene network structure, comparatively limited emphasis has been placed on interpreting the resulting large-scale gene networks.

Gene network enrichment analysis (GNEA) serves as a useful methodology for interpreting complex gene networks and elucidating the biological functions inherent in molecular interactions. GNEA aims to determine whether the topological features of a query network are statistically associated with relevant phenotypes. Alexeyenko *et al*. [4] enhanced traditional gene set enrichment analysis (GSEA) by incorporating gene–gene interaction networks, thereby developing a network enrichment approach that evaluates associations between gene sets based on network connectivity rather than simple gene overlap. Signorelli *et al*. [5] introduced the efficient network enrichment analysis test (NEAT), which extends conventional over-representation analysis by integrating gene–gene interaction networks and utilizing hypergeometric-based statistics to assess enrichment that reflects underlying network connections. Park *et al*. [6] developed gene behavior-based network enrichment analysis, which adapts differential gene expression analysis from GSEA into a network-based framework by considering gene behavior within the network. Although numerous GNEA frameworks have been developed to interpret complex gene regulatory networks, most current methods target binary phenotypes [5, 6]. In contrast, critical biological indicators, such as drug dose–response, stages of disease progression, or clinical physiological scales, are inherently continuous variables. Dichotomizing these continuous features results in considerable information loss, masking cellular heterogeneity and the subtle dynamics characteristic of biological systems. Existing methods for GNEA often fail to effectively score and rank associations with continuous phenotypes due to their inability to estimate molecular interactions corresponding to specific phenotypic values, thereby limiting the assessment of such associations [5, 6].

**Figure 1:**
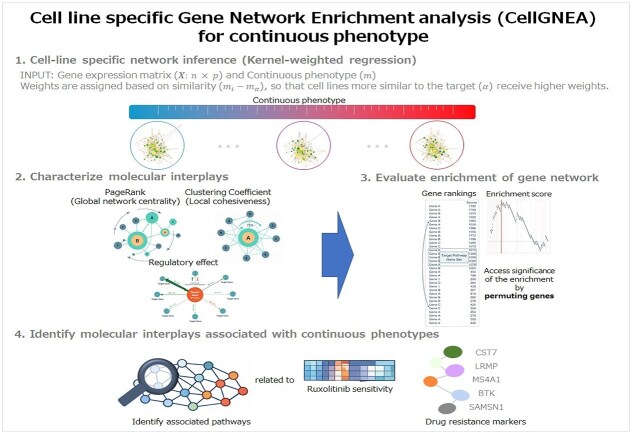
Overall framework of the CellGNEA. The input consists of gene expression data across multiple cell lines and a continuous phenotype. For each target cell line ($\alpha$), a cell line-specific gene network is estimated using a kernel-weighted regression model, where all samples are used and weighted according to their similarity in cell line characteristics. The estimated networks are then used to extract network-based features characterizing molecular interplays, followed by enrichment analysis to assess their association with the continuous phenotype via gene permutation. This framework enables the identification of molecular interactions and pathways associated with continuous phenotypic variation, addressing limitations of existing methods that rely on discrete group comparisons.

In the current study, we present a novel computational approach for GNEA, designated cell line-specific gene network enrichment analysis (CellGNEA). This methodology is designed to identify pathways with molecular interactions that are associated with continuous phenotypes. In GNEA, precise characterization of molecular interactions is essential, as they dictate the scores and rankings that underlie network enrichment assessments. Importantly, CellGNEA infers gene networks at the cell line level, integrating diverse network-derived metrics to characterize individual genes within each network context. Pathway-level associations are evaluated using a Kolmogorov–Smirnov (KS)-based statistic, with statistical significance determined using a permutation framework consistent with the principles of GSEA [7]. Our strategy also addresses a key limitation of existing enrichment analyses, highlighted by Tian *et al*. [8], namely that the conventional null distribution is constructed by permuting phenotype labels, implicitly assuming the absence of relevant genes within the query set. However, the corresponding hypothesis test is based on a KS statistic that assesses whether the association between the phenotype and a query gene set is stronger than that observed for other gene sets. This comparison is conceptually closer to gene-set permutation rather than phenotype permutation, leading to a mismatch between the test statistic and the assumed null model. Our approach resolves this issue by assessing pathway-level associations via KS statistics, with significance evaluated through gene-based rather than phenotype-based permutations. By permuting genes rather than phenotype labels, our platform enables rigorous testing of whether the molecular interplays in a query network demonstrate a uniquely significant association pattern relative to a null model comprising randomly permuted networks. Figure 1 illustrates the overall framework of the proposed CellGNEA. Several methods have been proposed to infer cell line-specific gene regulatory networks by leveraging either population-level or single-cell data. For example, LIONESS: Linear Interpolation to Obtain Network Estimates for Single Samples reconstructs individual networks by decomposing an aggregate network using a linear interpolation strategy based on leave-one-out perturbations [9]. In contrast, Bayesian Optimized sample-specific Networks Obtained By Omics data (BONOBO) adopts a probabilistic framework, modeling each sample-specific network as a deviation from a shared global structure and estimating edge weights within a Bayesian setting [10]. Furthermore, SWEET: Sample-specific network inference method infers individual networks by reweighting the contribution of each sample during the network reconstruction process, thereby emphasizing sample-specific effects [11]. While the weighting strategy in SWEET shares conceptual similarities with local kernel-based approaches in that it prioritizes sample-specific contributions, it does not explicitly formulate a localized optimization problem with sparsity-inducing regularization, as in kernel-based $L_{1}$-type regularized regression frameworks. In contrast to these population-based approaches, cell-specific network constructed by single-cell RNA sequencing data directly constructs cell-specific networks by evaluating gene–gene dependencies within each individual cell using local statistical measures derived from single-cell RNA sequencing data [12]. Although numerous strategies have been proposed to infer cell line-specific gene networks and capture heterogeneous molecular interplays underlying complex disease mechanisms, these approaches are fundamentally designed for network inference rather than functional interpretation. As a result, despite their ability to reconstruct individual-level networks, they lack a principled framework for linking the inferred networks to phenotypic variation or for performing pathway-level enrichment analysis. To the best of our knowledge, CellGNEA is the first computational strategy that enables the functional analysis of cell line-specific gene networks and facilitates gene network enrichment analysis with respect to continuous phenotypes. The overall framework of CellGNEA is illustrated in Fig. 1.

To systematically evaluate the performance of the proposed framework, we conducted Monte Carlo simulations. These simulations were designed to generate cell line-specific gene networks, and the outcomes demonstrate the robustness, statistical soundness, and enhanced power of our method in detecting network enrichment driven by continuous phenotypic variation. Furthermore, CellGNEA was used to identify molecular interactions associated with drug sensitivity within leukemia-related pathways. Our results demonstrated that the molecular interplays involved in the acute myeloid leukemia (AML) pathways were significantly associated with the response to doxorubicin, imatinib, quizartinib, and ruxolitinib. Notably, imatinib, quizartinib and ruxolitinib were consistently associated with molecular interplays across AML, myelodysplastic syndromes (MDS), and chronic myelogenous leukemia (CML), suggesting shared underlying network mechanisms. Our results highlight the potential resistance-associated genes PTPN11, MS4A1, and BTK, suggesting their roles as resistance-associated markers within leukemia-related mechanisms. The results of this study substantiate ability of CellGNEA to capture phenotype-associated, cell-line-specific rewiring at the pathway level. Our strategy provides insights beyond traditional gene-level analyses and emphasizes the importance of network-aware enrichment analysis for characterizing the biological attributes of cell lines.

The structure of this paper is as follows. The Material and methods section details the proposed CellGNEA framework. The Monte Carlo simulation section reports the outcomes of comprehensive simulation studies. Subsequent sections describe the analytical results pertaining to drug sensitivity-related molecular interactions within leukemia-related pathways. The Discussion section summarizes the conclusions.

## Materials and methods

The primary objective of this study was to identify functional pathways enriched for gene networks exhibiting regulatory properties associated with continuous phenotypes. Specifically, the following hypothesis was tested:

**Null hypothesis**: *Molecular interactions within a query gene network do not exhibit cell-line-specific association patterns with the continuous phenotype that differ from those observed in other gene networks.*

### Cell line-specific gene network inference

In this study, the input data consist of (i) gene expression profiles measured across multiple cell lines and (ii) a continuous modulator representing cell line-specific characteristics, such as drug sensitivity. The output of this stage is a set of cell line-specific gene networks, where each network describes regulatory relationships among genes tailored to an individual cell line. Suppose $\boldsymbol{X}=(\boldsymbol{x}_{1},...,\boldsymbol{x}_{n})^{T} \in \mathbb{R}^{n\times p}$ is $n \times p$ data matrix that describe the expression levels of $p$ genes for $n$ cell lines. The following linear regression framework was used to model the gene network,


(1) \begin{eqnarray*} x_{i\ell}=\sum_{j \neq \ell}^{p} \beta_{\ell j}x_{ij}+\epsilon_{i\ell}, \quad \ell=1,...,p,\quad i=1,\cdots,n,\end{eqnarray*}

where $x_{i\ell }$ is the expression level of the $\ell$th gene in the $i$th cell line, and $\boldsymbol{\varepsilon }_{\ell } = (\epsilon _{1\ell }, \dots , \epsilon _{n\ell })^{T} \in \mathbb{R}^{n}$ is a random error vector accounting for residual variation. The regression coefficient vector $\boldsymbol{\beta }_{\ell } = (\beta _{\ell 1}, \dots , \beta _{\ell -1}, \beta _{\ell +1}, \cdots \beta _{\ell p})^{T}$ indicates the edge weight of the $\ell$th target gene from other $p-1$ genes. Although regression-based frameworks are widely used to model molecular interactions and provide intuitive interpretability, they cannot capture cell line-specific interactions, as the estimated edge weights ($\boldsymbol{\beta }_{\ell }$) represent averages across $n$ cell lines rather than cell line-specific effects.

The cell line-specific gene networks are designed to capture molecular interactions that vary according to the characteristics of individual cell lines. To infer such networks, we introduce a cell line-specific characteristic variable $m_{\alpha }$ for $\alpha =1,...,n$ referred to as a modulator (e.g. drug sensitivity or disease progression). We model gene–gene regulatory relationships using a varying coefficient framework [13], in which regression coefficients are allowed to vary as functions of a modulator. Under this framework, the regulatory effect between the $\ell$th gene and $j$th gene is expressed as a function of the modulator value $m_{\alpha }$, denoted by $\beta _{\ell j}(m_{\alpha })$. That is, the coefficient functions $\beta _{\ell j}(m_{\alpha })$ are assumed to be smooth with respect to the modulator $m_{\alpha }$, allowing gene regulatory relationships to vary continuously across cell line characteristics. In addition, we assume that the underlying gene regulatory network is sparse, such that only a subset of genes regulates each target gene. For each cell line $\alpha$, the coefficient is evaluated at $m_{\alpha }$, yielding $\beta _{\ell j}(m_{\alpha })$. The varying coefficient model to infer gene network of $\alpha$th cell line is given as follows:


(2) \begin{eqnarray*} x_{i\ell}=\sum_{j \neq \ell}^{p} \beta_{\ell j}(m_{\alpha})x_{ij}+\epsilon_{i\ell}, \quad\end{eqnarray*}

where $\boldsymbol{\beta }_{\ell }(m_{\alpha }) = (\beta _{\ell 1}(m_{\alpha }), \ldots , \beta _{\ell p}(m_{\alpha }))^{T}$ denotes a varying coefficient vector that characterizes the strengths of the regulatory effects of $p-1$ genes on the $\ell$th target gene in the network of the $\alpha$th cell line, and $m_{\alpha }$ represents a biological characteristic of the $\alpha$th cell line. The cell-line specific gene networks (i.e. $\hat{\boldsymbol{\beta }}_{\ell }(m_{\alpha })$) were estimated using the following kernel-based $L_{1}$-type regularization method [14],


\begin{align*} & \hat{\boldsymbol{\beta}}_{\ell\alpha}=\mathop{\text{arg min}}\limits_{\boldsymbol{\beta}_{\ell\alpha}}\left\{\frac{1}{2}\sum^{n}_{i=1}\left(x_{i\ell}-\sum_{j \neq \ell}^{p} \beta_{\ell j\alpha}x_{ij}\right)^{2}K(m_{i}, m_\alpha \mid b_{\ell \alpha})\right.\\& \left.\qquad \ \ +\ P(|\boldsymbol{\beta}_{\ell\alpha}|)\right\}, \end{align*}

where $\boldsymbol{\beta }_{\ell \alpha }=\boldsymbol{\beta }_{\ell }(m_{\alpha })$ and $P(|\boldsymbol{\beta }_{\ell \alpha }|)$ denote the $L_{1}$-type penalty, such as the elastic net penalty term [15],


(3) \begin{eqnarray*} P(|\boldsymbol{\beta}_{\ell\alpha}|)\}=\lambda_{\ell \alpha}\sum_{j=1}^{p}\left[\frac{1}{2}(1-\pi_{\ell \alpha})\beta_{\ell j\alpha}^{2}+\pi_{\ell \alpha}|\beta_{\ell j\alpha}|\right],\end{eqnarray*}

where $\lambda _{\ell \alpha }>0$ is a regularization parameter that controls the degree of shrinkage for $\boldsymbol{\beta }_{\ell \alpha }$, $0 \leq \pi _{\ell \alpha } \leq 1$ is a mixing parameter between the $L_{2}$-norm [16] and $L_{1}$-norm [17] penalties, and $K(m_{i}, m_\alpha \mid b_{\ell \alpha })$ is a Gaussian kernel function,


(4) \begin{eqnarray*} K(m_{i}, m_\alpha \mid b_{\ell \alpha}) = \exp\left\{-\frac{(m_{i} - m_\alpha)^{2}}{b_{\ell \alpha}}\right\}\end{eqnarray*}

where $b_{\ell \alpha }$ is the bandwidth, and the kernel is defined based on the distance between two inputs $m_{i}$ and $m_\alpha$. In cell line-specific gene network estimation, the Gaussian kernel function serves a critical function by quantifying the similarity among cell line characteristics, measured as $(m_{i} - m_{\alpha })^{2}$, and determining the weight assigned to each cell line during gene network estimation of the $\alpha$th cell line. Thus, the gene network of the $\alpha$th cell line ($\alpha = 1, \ldots , n$) can be estimated by leveraging data predominantly from cell lines that exhibit characteristics (i.e. $m_{i}$ for $i=1,\cdots n$) that closely align with those of the $\alpha$th cell line (i.e. $m_{\alpha }$). Thus, the gene network of the $\alpha$th cell line ($\alpha = 1, \ldots , n$) can be estimated by leveraging data predominantly from cell lines that exhibit characteristics (i.e. $m_{i}$ for $i=1,\cdots n$) that closely align with those of the $\alpha$th cell line (i.e. $m_{\alpha }$). Notably, the $\alpha$th sample itself receives the highest weight in this kernel-based framework, while other samples contribute proportionally according to their similarity. Therefore, the gene network for the $\alpha$th cell line is estimated using its own data together with information borrowed from similar cell lines, improving estimation stability in high-dimensional settings.

We consider the following Bayesian Information Criterion (BIC, [18]) to select the regularization parameters (i.e. $\lambda _{\ell \alpha }$ and $\pi _{\ell \alpha }$) and the bandwidth $b_{\ell \alpha }$,


(5) \begin{eqnarray*}\mathrm{BIC}=\frac{||\boldsymbol{x}_{\ell}-\hat{\boldsymbol{x}}_{\ell}||^{2}}{n \sigma^{2}}+\frac{{\mathrm{log}(n)}}{n}\hat{df},\end{eqnarray*}

where $\hat{df}$ is the degree of freedom of the estimated model. We evaluate the BIC over a range of candidate values and select the optimal regularization parameters and bandwidth that minimize the BIC.

### Cell line-specific gene network enrichment analysis

To evaluate associations between the gene network and continuous phenotypes, we characterized cell-line-specific molecular interactions using several network analysis-derived features. These features were subsequently scored, and the null hypothesis was tested with a KS test [6, 7].

#### A novel association measure between gene network and continuous phenotype

For continuous phenotypes, GNEA requires characterizing molecular interactions within each cell line-specific network, as regulatory relationships may differ across cell lines and capture phenotype-dependent variations that cannot be represented by a single aggregated network. The properties of these cell line-specific molecular interactions were delineated according to the clustering coefficient [19], PageRank [20], and regulatory effect.

1. **Clustering coefficient (local cohesiveness)**
  The functional importance of genes within their sub-networks was evaluated by measuring the following clustering coefficient [19],
  (6) \begin{eqnarray*} C_{j\alpha}=\frac{\frac{1}{2}\big[\big(\boldsymbol{W}_{\alpha}+\boldsymbol{W}^{T}_{\alpha})(\boldsymbol{A}_{\alpha}+\boldsymbol{A}^{T}_{\alpha}\big)^{2}\big]_{jj}}{s^{\mathrm{tot}}_{j\alpha}(d^{\mathrm{tot}}_{j\alpha}-1)-2s^{\leftrightarrow}_{j\alpha}},\end{eqnarray*}
  where $0\leq C_{j\alpha }\leq 1$ and $[M]_{jj}$ represents the $(j,j)$th entry (i.e. the diagonal element corresponding to the $j$th gene) of matrix $M$. The $\boldsymbol{W}_{\alpha }$ and $\boldsymbol{A}_{\alpha }$ are the weighted adjacency matrix and the adjacency matrix, respectively, and
  \begin{align*} &s^{\mathrm{tot}}_{j\alpha}=\big(\boldsymbol{A}^{T}_{\alpha}\boldsymbol{W}_{\alpha}+\boldsymbol{A}_{\alpha}\boldsymbol{W}^{T}_{\alpha}\big)_{jj}=\big(\boldsymbol{W}^{T}_{\alpha}+\boldsymbol{W}_{\alpha}\big)\cdot\boldsymbol{1},\\ &d^{\mathrm{tot}}_{j\alpha}=\big(\boldsymbol{A}^{T}_{\alpha}+\boldsymbol{A}_{\alpha}\big)_{j}\cdot\boldsymbol{1},\\ &s^{\leftrightarrow}_{j\alpha}=\frac{1}{2}(\boldsymbol{W}_{\alpha}\boldsymbol{A}_{\alpha}+\boldsymbol{A}_{\alpha}\boldsymbol{W}_{\alpha})_{j}, \end{align*}
  where $\boldsymbol{1}$ is the unit column vector of the $p$ elements. The clustering coefficient $C_{j\alpha }$ measures the density of local neighborhoods by quantifying the weighted abundance of triangular interactions among the neighbors of the $j$th gene, normalized by its maximum possible connectivity. A high $C_{j\alpha }$ value indicated strong local cohesiveness and potential functional importance of the $j$th gene in the gene network of the $\alpha$th cell line.
2. **PageRank (global centrality)**
  We also considered global network centrality to capture overall regulatory influence of each gene. In contrast to the clustering coefficient, the following PageRank quantifies global topological influence by recursively propagating importance through neighboring nodes [20],
  (7) \begin{eqnarray*} \mathrm{PR}_{j\alpha}=(1-d)+d\sum_{k \in V_{j\alpha}}\frac{\mathrm{PR}_{k\alpha}}{L_{k\alpha}},\end{eqnarray*}
  where $0\leq \mathrm{PR}_{j\alpha } \leq 1$, $V_{j\alpha }$ is a set of nodes directly connected to the $j$th gene, $L_{k\alpha }$ is the number of links (i.e. edges) extending from gene $k$th in the network of the $\alpha$th cell line, and $d$ is the damping factor parameter (typically set to 0.85 [20]). The PageRank score in (7) is defined as the stationary solution of the recursive equation and are computed iteratively until convergence. The convergence to a unique solution is guaranteed for $0 < d < 1$, as the resulting transition matrix becomes irreducible and aperiodic. A high PageRank score suggests that a gene exerts strong global regulatory influence, playing a critical role in maintaining network stability and facilitating information propagation.
3. **Regulatory effects**
  The regulatory effect of the $j$th gene on the $\ell$th gene in the $\alpha$th cell line was defined as follows:
  (8) \begin{eqnarray*} \mathrm{RE}_{j\ell\alpha}=\hat{\beta}_{j\ell}(m_{\alpha})\cdot x_{\alpha j}.\end{eqnarray*}
  Thus, the total regulatory effect of the $j$th gene in the network of the $\alpha$th cell line was measured by
  (9) \begin{eqnarray*} \mathrm{RE}_{j\alpha}=x_{\alpha j}\sum_{\ell=1}^{p}|\hat{\beta}_{j\ell}(m_{\alpha})|.\end{eqnarray*}
  The regulatory effect represents the impact of the $j$th gene on the network, as variations in the expression level of the $j$th gene propagate to its target genes according to the associated edge weights.
4. **Cell line-specific gene features**
  We proposed a statistic to characterize gene activity within the cell line-specific gene network by integrating diverse network-derived information,
  (10) \begin{eqnarray*} z_{j\alpha}=\mathrm{RE}_{j\alpha}\left(\frac{C_{j\alpha}+\mathrm{PR}_{j\alpha}}{2}\right).\end{eqnarray*}
  The statistic $z_{j\alpha }$ represents a weighted regulatory effect defined by $C_{j\alpha }$ and $\mathrm{PR}_{j\alpha }$.
  To capture a comprehensive information of gene importance within cell line-specific networks, CellGNEA incorporates three complementary metrics: clustering coefficient, PageRank, and regulatory effects. The clustering coefficient is selected to measure local cohesiveness, identifying genes that function as critical components within dense functional modules or sub-networks. In contrast, PageRank is utilized to assess global centrality, quantifying a gene’s overall regulatory influence and its role in maintaining network stability and information flow across the entire system. While these topological metrics define the position of a gene, the regulatory effect provides a measure of biological signal impact by integrating gene expression with the estimated strength of regulatory interactions. These three metrics are synergistically integrated into a single statistic in (10), where the average of the topological metrics (local and global importance) serves as a weight for the functional regulatory effect. This multivariate characterization ensures that the final enrichment score reflects both the physical architecture of the network and the functional intensity of molecular interactions. By avoiding reliance on a single network descriptor, this integrated approach allows for more sensitive and robust detection of network-level remodeling associated with continuous phenotypes compared with traditional methods.
5. **A novel association measure**
  To evaluate the association between the cell line-specific gene networks and continuous phenotypes, we assessed how the characteristics of molecular interactions ($z_{j\alpha }$ for $\alpha =1,...,n$) vary depending on the continuous phenotype ($m_{\alpha }$ for $\alpha =1,...,n$). That is, the association between gene networks with continuous phenotypes was assessed based on correlations between $\boldsymbol{z}_{j}=(z_{j1},\cdots z_{jn})$ and the modulator $\boldsymbol{m}=(m_{1},\cdots m_{n})$ describing the continuous phenotypic values,
  (11) \begin{eqnarray*} \gamma_{j}=\frac{\sum_{i=1}^{n}(m_{i}-\bar{m})(z_{ij}-\bar{z}_{j})}{\sum_{i=1}^{n}(m_{i}-\bar{m})^{2}(z_{ij}-\bar{z}_{j})^{2}}, \quad j \in V,\end{eqnarray*}
  where $V$ is the set of nodes comprising a query network, $\bar{m}$ and $\bar{z}_{j}$ are the means of the modulator values (i.e. phenotypic values) and $j$th gene activity across $n$ cell lines, respectively.

#### Enrichment score of the query network for continuous phenotypes

The enrichment score evaluates the overrepresentation of genes involved in a specific pathway ($j \in V^{*}$) at the top or bottom of the ranking list induced by $r_{j}$ across all genes in the query network ($j \in V$) in line with [7].

1. All genes were ranked in the query network (i.e. $j \in V$) to form an ordered gene list $R = \{v_{(1)}, \ldots , v_{(p)} \}$ according to the statistic $\gamma _{j}$.
2. A running-sum statistic was computed over the ranking list $R$ by increasing the score when a gene $v_{(i)} \in V^{*}$ was encountered (“Hit”), weighted by $\gamma _{v_{(i)}}$; otherwise the score was decreased (“Miss”),
  \begin{align*} &\mathrm{Hit}(V^{*},i)=\sum_{v_{(j)\in V^{*}},j \leq i}\frac{|\gamma_{j}^{k}|}{V^{0}}, \quad\mathrm{where} \quad V^{0}=\sum_{v_{(j)}\in V^{*}}|\gamma_{j}|^{k}\\ &\mathrm{Miss}(V^{*},i)=\sum_{v_{(j)}\notin V^{*}, j\leq i}\frac{1}{|V|-|V^{*}|}, \end{align*}
  where $|V|$ denotes the number of genes belonging to node set $V$. The enrichment score $eSC$ was defined as the maximum deviation from zero of the cumulative sum of $\mathrm{Hit}(V^{*}, i) - \mathrm{Miss}(V^{*}, i)$, as $i$ ranges over $V$. During this process, the score increases incrementally if the ranked gene $v_{(i)} \in R$ belongs to the $V^{*}$ and decreases otherwise. Conceptually, $eSC$ can be viewed as a weighted extension of the KS statistic that becomes the classical unweighted KS statistic when $k=0$ [7].

#### Significance assessment of the enrichment score

We evaluated the significance of the $eSC$ using the permutation framework. Tian *et al*. [8] highlighted a central inconsistency in the GSEA framework. That is, in the GSEA [7], the null distribution was constructed by permuting phenotype labels, assuming that the query gene set contained no genes associated with the phenotype of interest. In contrast, hypothesis testing employed a KS statistic to determine whether a given gene set exhibited a stronger association with the phenotype than other sets, assessing statistical significance against a null distribution generated by gene permutation rather than phenotype permutation. This approach resulted in a misalignment between the null distribution assumption and the hypothesis being tested, leading to a conceptual disparity between the test statistic and the inferential framework. To address the fundamental inconsistency and accurately test our hypothesis “Molecular interplays within a query gene network do not exhibit cell line-specific association patterns with the continuous phenotype that differ from those observed in other gene networks,” we proposed a framework to evaluate the enrichment significance by permuting genes within the network, rather than phenotype labels. This enables a more accurate assessment of the statistical significance of gene network enrichment.

1. For each permutation $\omega = 1, \ldots , \Omega$, a permuted pathway gene set $V^{*}_{\omega }$ is implicitly defined by randomly permuting the genes from $V$.
2. We then recomputed $eSC(V^{*}_{\omega })$ and normalized the scores by separately rescaling positive and negative values using the mean of the corresponding permuted scores, yielding the normalized enrichment scores, NeSC$(V^{*})$ and NeSC$(V^{*}_{\omega })$.
3. Compute permutation $P$-value
  (12) \begin{eqnarray*} P\text{ -}value=\frac{\sum_{\omega=1}^{\Omega}\mathbb{I}({\text{ NeSC}}(V^{*})\leq{\mathrm{NeSC}}(V^{*}_{\omega}))}{\Omega},\end{eqnarray*}
  where $\mathbb{I}$ is an indicator function. We considered the query network statistically significant when $P$-value was less than the significance level.
4. To account for multiple pathway-level tests in network enrichment analysis, we controlled the false discovery rate by applying the Benjamini–Hochberg procedure [21].

## Results

### Monte Carlo Simulations 1: Evaluation under idealized conditions

The efficacy of the proposed CellGNEA was evaluated through Monte Carlo simulation experiments designed to assess whether CellGNEA can identify pathway-level molecular interactions associated with continuous phenotypic variation under cell line-specific network heterogeneity. The detailed simulation framework is described below. For each simulated cell line $\alpha = 1, \ldots , n$, a weighted adjacency matrix $\mathbf{W}_{\alpha } \in \mathbb{R}^{p \times p}$ was generated as follows:


(13) \begin{eqnarray*} \boldsymbol{W}_{\alpha} = \big(w^{(\alpha)}_{ij}\big)_{1 \le i,j \le p}, \quad w^{(\alpha)}_{ij} = \begin{cases} w^{(\alpha)}_{ij}, & \mathrm{if}\ i < j, \\ w^{(\alpha)}_{ji}, & \mathrm{if}\ i> j, \\ 0, & \mathrm{if}\ i = j, \end{cases}\end{eqnarray*}

where the upper-triangular entries $w^{(\alpha )}_{ij}$ are independently sampled as $w^{(\alpha )}_{ij} \sim U(-5, 5), \quad \mathrm{for} \alpha = 1, \ldots , n,$ and the lower-triangular part is defined by symmetry, i.e. $w^{(\alpha )}_{ji} = w^{(\alpha )}_{ij}$. This approach yielded the edge weights differed among cell lines, capturing both positive and negative regulatory effects. The weighted adjacency matrices were used to arrange the gene network edge weights for $n$ cell lines into a structured matrix $\boldsymbol{\Theta }=[\boldsymbol{\theta }_{1},\cdots , \boldsymbol{\theta }_{n}]$, where $\boldsymbol{\theta }_{\alpha } \in \mathbb{R}^{p(p-1)/2}$ is a vector describing an edge weight of the $\alpha$th cell line. This design materialized cell-line-specific heterogeneity in edge strengths.

To construct molecular interactions associated with continuous phenotypes, we defined $q$ pathway-related genes and recalibrated their edge weights such that the edge strengths systematically increased or decreased as a function of the modulator values $\boldsymbol{m}$, denoted by $\boldsymbol{\Theta }^{\prime}$. That is, only edges of the $q$ pathway-related genes having positive edge weight $\kappa$ at the first cell line were assigned as a sequence of equally spaced values between $\kappa$ and $0$, i.e. $\left \{ \kappa - \frac{i}{n-1}\kappa \right \}_{i=0}^{n-1}$. For the negative edge weight $-\kappa$ at the first cell line, edge weights were assigned as a sequence of equally spaced values between $-\kappa$ and $0$, i.e. $\left \{ -\kappa + \frac{i}{n-1}\kappa \right \}_{i=0}^{n-1}$. The matrix $\boldsymbol{\Theta }^{\prime}$ in (14) illustrates an example of edge weight configurations for $n$ cell lines, in which the first and third genes are designated as pathway-related genes,


(14) \begin{eqnarray*} \boldsymbol{\Theta}^{\prime}= \begin{pmatrix} 5 & (5 - \frac{5}{n})& (5 - \frac{10}{n}) &\cdots & 0\\ 0 & 3 &1 &\cdots & 3\\ -3 & (-3 + \frac{3}{n} ) & (-3 + \frac{6}{n} ) &\cdots & 0\\ \vdots & \vdots& \vdots &\ddots& \vdots\\ -2& 1&-2&\cdots & -3\\ \end{pmatrix}.\end{eqnarray*}

Next, we generated the expression levels of genes $\boldsymbol{X}$ under the following structural equation models [22, 23],


(15) \begin{eqnarray*} \boldsymbol{X}=\boldsymbol{W}_{\alpha}\boldsymbol{X}+\boldsymbol{E}_{\alpha},\end{eqnarray*}

where $\boldsymbol{E}_{\alpha } \in \mathbb{R}^{n\times p}$ is a noise matrix with entries independently drawn from a Gaussian distribution with mean zero and variance $\sigma ^{2}$. That is, the expression levels of the genes were generated by the constructed weighted adjacency matrix $\boldsymbol{W}_{\alpha }$ from the edge weights $\boldsymbol{\theta }_{\alpha }$ as follows:


(16) \begin{eqnarray*} \boldsymbol{X}=(\boldsymbol{I}-\boldsymbol{W}_{\alpha})^{-1}\boldsymbol{E}_{\alpha}.\end{eqnarray*}

For the $q$ pathway-related genes, we regenerated expression levels based on a continuous phenotype as follows:


(17) \begin{eqnarray*} \boldsymbol{x}^{\prime}_{j}=\boldsymbol{x}_{j}+\mathbb{I}(j \in \mathcal{S}) \, \tilde{\boldsymbol{m}},\quad j \in \mathcal{P},\end{eqnarray*}

where $\mathcal{P}$ represents the set of pathway-related genes. A subset $\mathcal{S} \subset \mathcal{P}$ of signal genes was selected from $\rho \%$ of pathway-related genes, where $\rho \sim \mathrm{Unif}(0.25, 0.75)$. The term $\tilde{\boldsymbol{m}}$ denotes the standardized modulator. The indicator function $\mathbb{I}(\cdot )$ ensures that only the selected signal genes are modulated by the phenotype-driven signal. We denoted the reconstructed expression levels of $q$ pathway-related genes by $\boldsymbol{X}^{\prime}$. To rigorously evaluate CellGNEA’s capability in capturing dynamic biological transitions, our simulation design mirrors two critical features of the problem setting: (i) continuous phenotypic gradients and (ii) cell line-specific network rewiring. Unlike static benchmarks, this approach ensures that the simulated data reflect the heterogeneous biological systems where regulatory interactions evolve alongside phenotypes. The simulation framework is specifically designed to reflect the inherent complexities of cell line-specific biological systems. Equations (14) and (15) represent the structural connectivity of the gene network, where $\boldsymbol{W}_{\alpha }$ captures the unique regulatory topology and heterogeneous rewiring of each individual cell line ($\alpha$). Furthermore, Equation (16) models the phenotype-driven signal, ensuring that the network enrichment is not merely a statistical artifact of random gene expression but a systematic, biological response to the continuous modulator ($m$) representing the phenotypic gradient.

We defined a true-positive scenario for GNEA by generating network structures $\boldsymbol{\Theta }^{\prime}$ and gene expression levels $\boldsymbol{X}^{\prime}$ by incorporating pathway-related genes. In contrast, for the true-negative scenario, edge weights $\boldsymbol{\Theta }$ and expression levels $\boldsymbol{X}$ were created without incorporating pathway-related gene information. Subsequently, we applied our strategy to the gene networks containing $p=250, 500,$ and $1000$ genes, with pathway-related gene proportions of 5% and 25%. To evaluate the effectiveness of CellGNEA, we compared our strategy with several existing approaches. These include ORA [24] as a gene-list-based enrichment approach representing first-generation pathway analysis methods [25], GSEAn and GSEAc [26] because they incorporate pathway topology and network centrality information into enrichment analysis, consistent with topology-aware analytical strategies summarized in previous pathway analysis reviews [25, 27], and NEAT [5] because it incorporates network information into enrichment analysis. Comparator methods were selected to cover different analytical strategies relevant to pathway enrichment analysis, including gene-list-based enrichment, topology-aware enrichment, and network-based enrichment approaches. Our objective was to evaluate CellGNEA across multiple analytical strategies relevant to pathway enrichment analysis rather than exhaustively compare every available methodology. Therefore, not all available methodologies were included in the comparison. For example, clusterProfiler [28] was not included because it implements an over-representation analysis framework already represented by ORA, TopologyGSA [29] was not included because it incorporates pathway topology information similar to that considered by GSEAn and GSEAc, and EnrichNet [30] was not included because it evaluates functional associations based on network connectivity, an analytical strategy already represented by NEAT. This design enables evaluation of whether CellGNEA provides improved performance under continuous phenotypic settings while avoiding redundant comparisons among methods with similar analytical principles.

By including these diverse comparators, we ensure a comprehensive assessment against established benchmarks for both gene-set and network-based analysis. In contrast, CellGNEA utilizes a kernel-based varying coefficient model to estimate phenotype-specific edge weights across a continuous spectrum. Based on a review of recent literature, including pathway and network enrichment analysis reviews [31–33], and a PubMed and Google Scholar search conducted in June 2026 using combinations of the keywords “gene network enrichment analysis,” “network enrichment analysis,” “continuous phenotype,” “continuous outcome,” and “pathway enrichment,” we did not identify an established GNEA framework that directly integrates continuous phenotypes with gene-network-level pathway analysis. Therefore, we selected benchmark approaches developed for categorical phenotypes to assess whether CellGNEA addresses this methodological gap while providing improved performance in sensitive and heterogeneous settings. To further evaluate the robustness of CellGNEA under sample-specific network inference settings, we additionally evaluated a LIONESS-based approach using a leave-one-out strategy. LIONESS was selected because it has been introduced and evaluated as a sample-specific network inference framework in previous review and benchmarking studies [34, 35]. Since CellGNEA was developed as a gene network enrichment analysis framework rather than a sample-specific network inference method, the LIONESS-based analysis was performed to assess the sensitivity of CellGNEA to sample-specific network estimation results. To improve the stability of network estimation, we adopted a hybrid network inference strategy integrating regression-based and correlation-based networks, leveraging their complementary strengths in capturing conditional dependencies and stable co-expression patterns. The significance level was set to 0.05, and the number of permutations ($\Omega$) was set to 1000 to compute permutation-based $P$-values.

**Figure 2:**
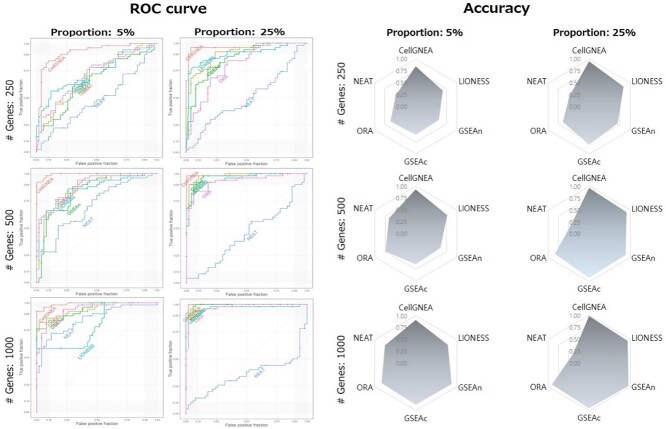
Accuracy (left) and ROC curve (right) of the gene network enrichment analysis evaluated using simulation studies. CellGNEA, Cell Line-Specific Gene Network Enrichment Analysis; LIONESS, Linear Interpolation to Obtain Network Estimates for Single Samples; GSEAn, Gene Set Enrichment Analysis with network structure; GSEAc, Gene Set Enrichment Analysis with centrality measure; ORA, Over-representation analysis; NEAT, network enrichment analysis test.

Figure 2 presents the receiver operating characteristic (ROC) curve and the accuracy of the cell-line-specific GNEA evaluated with a continuous phenotype. As shown in Fig. 2, methods based on cell line-specific gene network inference (i.e. CellGNEA and LIONESS) demonstrate superior performance, with the proposed CellGNEA consistently outperforming existing approaches. While NEAT exhibited limited performance, its effectiveness further decreased as the proportion of pathway-related genes increased. The observed accuracy in enriched network identification also supports the efficacy of our approach. Although the GSEA methods based on the network and centrality metrics (i.e. GSEAn and GSEAc) performed well in scenarios with numerous genes and a high proportion of pathway-related genes, their overall performance remains limited.

We also evaluated our strategy using several metrics, including true positive rate (TPR), true negative rate (TNR), accuracy (ACC), precision (PREC), recall (REC), and F1 score (F1score), as detailed in Table 1. Consistent with the findings presented in Fig. 2, approaches based on cell line-specific gene network inference (i.e. CellGNEA and LIONESS) consistently demonstrate competitive performance across multiple metrics and experimental scenarios, with CellGNEA showing superior performance overall. Conversely, GSEA methods (specifically, GSEAn and GSEAc) failed to accurately identify gene networks enriched for the pathway, as evidenced by their low TPR values, particularly when pathway-related genes comprised a small fraction of the dataset. Although most approaches were proficient at detecting non-enriched gene networks, NEAT was notably less effective in terms of TNRs.

**Table 1:** Performance evaluation of gene network enrichment analysis methods in Monte Carlo Simulation 1 across different numbers of genes (250, 500, and 1000) and proportions of perturbed genes (5% and 25%), with bold numbers denoting the best-performing method.

No. Genes	Methods	Proportion: 5%						Proportion: 25%					
		TPR	TNR	ACC	PREC	REC	F1score	TPR	TNR	ACC	PREC	REC	F1score
250	CellGNEA	0.74	0.96	**0.85**	0.95	0.74	0.83	0.94	0.96	**0.95**	0.96	0.94	0.95
	LIONESS	0.42	0.90	0.66	0.81	0.42	0.55	0.80	0.88	0.84	0.87	0.80	0.83
	GSEAn	0.18	0.96	0.57	0.82	0.18	0.30	0.48	0.92	0.70	0.86	0.48	0.62
	GSEAc	0.22	0.96	0.59	0.85	0.22	0.35	0.64	0.96	0.80	0.94	0.64	0.76
	ORA	0.28	0.96	0.62	0.88	0.28	0.42	0.34	0.94	0.64	0.85	0.34	0.49
	NEAT	0.22	0.76	0.49	0.48	0.22	0.30	0.50	0.44	0.47	0.47	0.50	0.49
500	CellGNEA	0.86	1.00	**0.93**	1.00	0.86	0.92	0.94	1.00	**0.97**	1.00	0.94	0.97
	LIONESS	0.62	0.92	0.77	0.89	0.62	0.73	0.90	0.92	0.91	0.92	0.90	0.91
	GSEAn	0.26	0.94	0.60	0.81	0.26	0.39	0.84	0.94	0.89	0.93	0.84	0.88
	GSEAc	0.36	0.94	0.65	0.86	0.36	0.51	0.90	0.96	0.93	0.96	0.90	0.93
	ORA	0.50	0.98	0.74	0.96	0.50	0.66	0.72	0.96	0.84	0.95	0.72	0.82
	NEAT	0.48	0.82	0.65	0.73	0.48	0.58	0.68	0.18	0.43	0.45	0.68	0.54
1000	CellGNEA	0.82	1.00	**0.91**	1.00	0.82	0.90	1.00	0.98	**0.99**	0.98	1.00	0.99
	LIONESS	0.58	0.98	0.78	0.97	0.58	0.73	0.92	0.96	0.94	0.96	0.92	0.94
	GSEAn	0.76	1.00	0.88	1.00	0.76	0.86	0.98	0.94	0.96	0.94	0.98	0.96
	GSEAc	0.78	0.98	0.88	0.98	0.78	0.87	0.98	0.94	0.96	0.94	0.98	0.96
	ORA	0.76	0.92	0.84	0.90	0.76	0.83	0.84	0.98	0.91	0.98	0.84	0.90
	NEAT	0.76	0.74	0.75	0.75	0.76	0.75	0.72	0.06	0.39	0.43	0.72	0.54
Average	CellGNEA	0.81	0.99	**0.90**	0.98	0.81	0.89	0.96	0.98	**0.97**	0.98	0.96	0.97
	LIONESS	0.54	0.93	0.74	0.89	0.54	0.67	0.87	0.92	0.90	0.92	0.87	0.89
	GSEAn	0.40	0.97	0.68	0.88	0.40	0.52	0.77	0.93	0.85	0.91	0.77	0.82
	GSEAc	0.45	0.96	0.71	0.89	0.45	0.57	0.84	0.95	0.90	0.95	0.84	0.88
	ORA	0.51	0.95	0.73	0.91	0.51	0.64	0.63	0.96	0.80	0.92	0.63	0.74
	NEAT	0.49	0.77	0.63	0.65	0.49	0.54	0.63	0.23	0.43	0.45	0.63	0.52

The results indicate that characterizing cell line-specific molecular interactions is crucial for functional pathway analysis associated with continuous phenotypes. Overall, these results suggest that the proposed strategy, CellGNEA, may provide a potentially useful framework for identifying functionally relevant pathways within complex gene interaction networks.

To further evaluate our strategy based on the gene permuting, we conducted an additional comparison between gene permutation and phenotype permutation under the same simulation setting ($p=500$). In the phenotype permutation-based approach, the association measure is computed using permuted phenotype values. Based on this, the corresponding permuted enrichment scores are obtained, and the permutation $P$-values are subsequently calculated. The results are summarized in Table 2. Overall, gene permutation consistently outperformed phenotype permutation across most evaluation metrics. In particular, gene permutation achieved higher true positive rates (TPR) and recall values in both settings (5% and 25% proportions of pathway-related genes), indicating improved sensitivity in detecting enriched networks. Although phenotype permutation exhibited slightly higher true negative rates (TNR) and precision in some cases, this improvement was accompanied by a reduction in recall. These findings demonstrate that gene permutation provides a more balanced performance, especially in terms of sensitivity, while maintaining competitive accuracy and F1 scores. Importantly, this empirical advantage is consistent with the theoretical motivation of our framework, which employs gene permutation to align the null model with the hypothesis being tested. This supports the validity of the proposed gene permutation-based strategy for capturing pathway-level associations under continuous phenotypes.

**Table 2:** Comparison of gene permutation and phenotype permutation strategies under the simulation setting ($p=500$).

Proportion (%)	Permuting	TPR	TNR	ACC	PREC	REC	F1 score
5	Genes	0.76	0.86	0.81	0.84	0.76	0.80
	Phenotype	0.72	0.82	0.77	0.80	0.72	0.76
25	Genes	0.90	0.88	0.89	0.88	0.90	0.89
	Phenotype	0.86	0.90	0.88	0.90	0.86	0.88

We also evaluated the computational efficiency of the proposed method by comparing it with existing approaches, including ORA, GSEA-based methods (GSEAn and GSEAc), NEAT, and LIONESS under identical simulation settings (Table 3). For the runtime analysis, we measured the total execution time (in seconds) required to analyze datasets with varying numbers of genes. The results show that CellGNEA requires longer computation time than the competing methods across all settings, reflecting the additional computational burden of kernel-weighted regression performed for each gene and sample. In particular, the runtime increases substantially as the number of genes grows, indicating higher computational complexity compared to simpler methods such as ORA and GSEA. For the memory usage analysis, we recorded the peak RAM consumption (in Gigabyte) during execution. In contrast to runtime, the memory usage of CellGNEA is comparable to that of other methods, with only modest increases observed as the dataset size grows. Across all settings, the peak memory consumption remained <2 GB, suggesting that the method is feasible on standard workstation environments without requiring high-performance computing resources. Overall, although CellGNEA incurs higher computational time, its memory requirements remain moderate, supporting its practical applicability. All computational experiments were conducted on a workstation equipped with an Intel Core i9-12900 processor (16 cores, base frequency 2.40 GHz) and 64 GB of system memory. All analyses were implemented in R and executed in a single-threaded environment. No GPU acceleration was used.

**Table 3:** Comparison of computational efficiency across methods under varying dataset sizes ($p=250, 500, 1000$). Runtime (in seconds) and peak memory usage (in Gigabyte) are reported for CellGNEA, LIONESS, ORA, GSEAn, GSEAc, and NEAT under identical simulation settings

Methods	No. Genes: 250		No.Genes: 500		No. Genes: 1000	
	Time	RAM	Time	RAM	Time	RAM
CellGNEA	287.79	0.54	815.84	0.80	1780.17	1.69
LIONESS	63.19	0.54	277.19	0.72	831.35	1.68
ORA	8.80	0.50	47.45	0.50	250.09	0.99
GSEAn	22.75	0.49	59.72	0.75	234.09	1.08
GSEAc	22.16	0.49	55.57	0.75	243.22	1.08
NEAT	8.47	0.49	40.03	0.75	234.70	1.08

### Monte Carlo Simulations 2: Evaluation under relaxed and realistic conditions

In the Monte Carlo Simulations 1, edge weights for pathway-related genes were defined as equally spaced linear sequences between $\kappa$ and $0$ (or $-\kappa$ and $0$), yielding deterministic and strictly monotonic trajectories across cell lines. To provide a more realistic evaluation, we modify the construction of the pathway-related components of $\boldsymbol{\Theta }^{\prime}$ by relaxing this deterministic structure. Specifically, the original edge-weight sequences defined in (14) are perturbed through two types of modifications: a nonlinear transformation and stochastic perturbations.

- First, a mild nonlinear transformation is applied to the original edge-weight sequences. Let $\theta ^{\prime}_{ik}$ denotes the original equally spaced edge-weight sequence defined in (14), given by $\theta ^{\prime}_{ik} = \kappa - \frac{i}{n-1}\kappa$ for positive edges and $\theta ^{\prime}_{ik} = -\kappa + \frac{i}{n-1}\kappa$ for negative edges. We then apply an element-wise nonlinear transformation:
  \begin{align*} \theta^{\prime\prime}_{ik} = \theta^{\prime}_{ik} (1 + \gamma |\theta^{\prime}_{ik}|), \end{align*}
  which amplifies larger edge-weight values more than smaller ones, thereby introducing curvature into the trajectories and deviating from the original linear pattern.
- Second, stochastic perturbations are introduced to incorporate sparsity and structural variability. Specifically, we apply a dropout mechanism by multiplying each element by an independent Bernoulli random variable,
  (18) \begin{eqnarray*} Z_{ik} \sim \mathrm{Bernoulli}(\tau),\end{eqnarray*}
  where $\tau = 0.8$, so that a subset of edge-weight values is set to zero with probability $1 - \tau = 0.2$, thereby inducing sparsity across the edge-weight sequences. In addition, to allow structural inconsistencies in regulatory direction, we introduce random sign perturbations by multiplying each edge-weight value by either $+1$ or $-1$, with a small probability of sign reversal (i.e. $0.05$).

These modifications yield edge-weight configurations that deviate from the deterministic linear sequences defined in (14). The resulting sequences exhibit nonlinear behavior, stochastic variability, sparsity, and structural inconsistencies. Consequently, the revised simulation departs from the original varying-coefficient structure and provides a more realistic and challenging evaluation setting.

**Figure 3:**
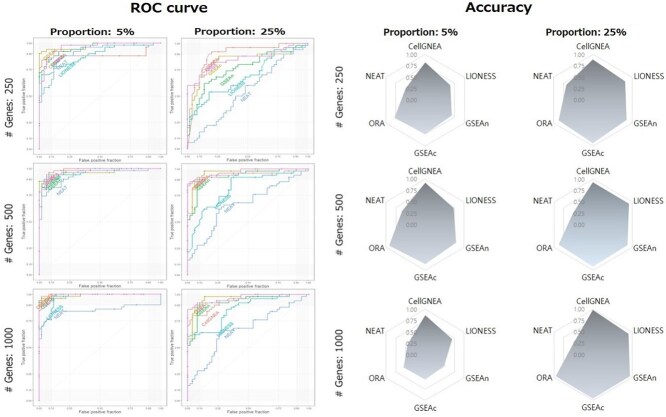
ROC curves (left) and accuracy (right) of gene network enrichment analysis methods evaluated under the revised simulation setting with relaxed assumptions. Results are presented across different gene sizes (250, 500, 1000) and proportions of pathway-related genes (5% and 25%). CellGNEA: Cell Line-Specific Gene Network Enrichment Analysis; LIONESS: Linear Interpolation to Obtain Network Estimates for Single Samples; GSEAn: Gene Set Enrichment Analysis with network structure; GSEAc: Gene Set Enrichment Analysis with centrality measure; ORA: Over-representation analysis; NEAT: network enrichment analysis test.

Under the realistic scenario, we evaluated our strategy. Figure 3 shows ROC curve and the accuracy of the cell-line-specific GNEA evaluated with a continuous phenotype. The overall performance of all methods decreases compared to the Monte Carlo Simulations 1 setting, reflecting the increased complexity introduced by nonlinear distortions, stochastic perturbations, sparsity, and structural inconsistencies. Despite this more challenging scenario, methods based on cell line-specific gene network inference continue to demonstrate competitive performance. In particular, the proposed CellGNEA consistently achieves robust performance across different network sizes (i.e. $p$) and proportions of pathway-related genes, indicating its stability under model misspecification and realistic data conditions.

In contrast to the Monte Carlo Simulations 1, where near-perfect performance was observed, the ROC curves and accuracy metrics in this setting no longer exhibit idealized behavior, suggesting that the revised simulation provides a more realistic assessment of method performance. While NEAT exhibits relatively limited performance across all scenarios, its degradation is more pronounced under the relaxed simulation setting. The GSEA-based methods (i.e. GSEAn and GSEAc) show moderate performance in some settings, particularly with larger gene sizes, but remain less consistent overall.

Table 4 presents a quantitative summary of the performance metrics under the revised simulation setting. Consistent with the results in Fig. 3, all methods exhibit increased false positive rates and reduced F1-scores compared to the Monte Carlo Simulations 1, reflecting the increased difficulty of the problem and a clear departure from the idealized conditions. Despite this, CellGNEA maintains a favorable balance between sensitivity and specificity across all scenarios, as evidenced by its relatively stable ACC and F1-scores. This indicates that the proposed method is robust to the introduced perturbations and remains reasonably stable in identifying pathway-related networks under more complex and realistic conditions. In contrast, competing methods show greater variability across different settings. The GSEA-based approaches (GSEAn and GSEAc) exhibit inconsistent performance depending on network size and pathway proportion. ORA generally achieves high specificity at the expense of reduced sensitivity, and NEAT shows consistently weaker performance across multiple evaluation metrics.

**Table 4:** Results of gene network enrichment analysis in Monte Carlo Simulation 2. Performance evaluation under Monte Carlo Simulation 2 across different numbers of genes (250, 500, and 1000) and proportions of perturbed genes (5% and 25%). Performance was evaluated using true positive rate (TPR), true negative rate (TNR), accuracy (ACC), precision (PREC), recall (REC), and F1 score (F1score). TPR represents the proportion of truly enriched pathways correctly identified, TNR indicates the proportion of non-enriched pathways correctly excluded, ACC denotes overall classification accuracy, PREC measures the proportion of correctly identified enriched pathways among predicted positives, REC measures sensitivity in detecting enriched pathways, and F1 score summarizes the balance between precision and recall. Higher values indicate better performance

No. Genes	Methods	Proportion: 5%						Proportion: 25%					
		TPR	TNR	ACC	PREC	REC	F1score	TPR	TNR	ACC	PREC	REC	F1score
250	CellGNEA	0.82	0.84	**0.83**	0.84	0.82	0.83	0.84	0.94	0.89	0.93	0.84	0.88
	LIONESS	0.42	0.86	0.64	0.75	0.42	0.54	0.78	0.86	0.82	0.85	0.78	0.81
	GSEAn	0.46	0.90	0.68	0.82	0.46	0.59	0.74	0.98	0.86	0.97	0.74	0.84
	GSEAc	0.56	0.92	0.74	0.88	0.56	0.68	0.90	0.98	**0.94**	0.98	0.90	0.94
	ORA	0.66	0.90	0.78	0.87	0.66	0.75	0.82	0.94	0.88	0.93	0.82	0.87
	NEAT	0.10	0.90	0.50	0.50	0.10	0.17	0.98	0.38	0.68	0.61	0.98	0.75
500	CellGNEA	0.86	0.98	**0.92**	0.98	0.86	0.91	0.94	0.92	**0.93**	0.92	0.94	0.93
	LIONESS	0.62	0.84	0.73	0.79	0.62	0.70	0.88	0.96	0.92	0.96	0.88	0.92
	GSEAn	0.64	0.94	0.79	0.91	0.64	0.75	0.88	0.90	0.89	0.90	0.88	0.89
	GSEAc	0.78	0.96	0.87	0.95	0.78	0.86	0.88	0.96	0.92	0.96	0.88	0.92
	ORA	0.86	0.98	**0.92**	0.98	0.86	0.91	0.80	0.96	0.88	0.95	0.80	0.87
	NEAT	0.26	0.92	0.59	0.76	0.26	0.39	1.00	0.02	0.51	0.51	1.00	0.67
1000	CellGNEA	0.78	0.96	**0.87**	0.95	0.78	0.86	0.98	0.98	**0.98**	0.98	0.98	0.98
	LIONESS	0.66	0.72	0.69	0.70	0.66	0.68	0.94	0.88	0.91	0.89	0.94	0.91
	GSEAn	0.00	0.96	0.48	0.00	0.00	0.00	0.90	1.00	0.95	1.00	0.90	0.95
	GSEAc	0.14	0.94	0.54	0.70	0.14	0.23	0.92	1.00	0.96	1.00	0.92	0.96
	ORA	0.16	0.94	0.55	0.73	0.16	0.26	0.90	1.00	0.95	1.00	0.90	0.95
	NEAT	0.16	0.74	0.45	0.38	0.16	0.23	1.00	0.00	0.50	0.50	1.00	0.67
Average	CellGNEA	0.82	0.93	**0.87**	0.92	0.82	0.87	0.92	0.95	**0.93**	0.94	0.92	0.93
	LIONESS	0.57	0.81	0.69	0.75	0.57	0.64	0.87	0.90	0.88	0.90	0.87	0.88
	GSEAn	0.37	0.93	0.65	0.58	0.37	0.45	0.84	0.96	0.90	0.96	0.84	0.89
	GSEAc	0.49	0.94	0.72	0.84	0.49	0.59	0.90	0.98	0.94	0.98	0.90	0.94
	ORA	0.56	0.94	0.75	0.86	0.56	0.64	0.84	0.97	0.90	0.96	0.84	0.90
	NEAT	0.17	0.85	0.51	0.55	0.17	0.26	0.99	0.13	0.56	0.54	0.99	0.70

Importantly, although our proposed method does not consistently yield superior performance across all scenarios as observed in Monte Carlo Simulations 1, the revised setting reveals more nuanced differences among the methods, with performance varying depending on the specific conditions.

### Functional pathway analysis of the leukemia drug sensitivity-specific gene networks

We selected leukemia as an application domain due to its well-established molecular and clinical heterogeneity, which gives rise to a continuous spectrum of drug responses rather than discrete subgroups [36, 37]. This characteristic makes leukemia particularly suitable for investigating phenotype-dependent rewiring of molecular interactions beyond conventional dichotomized analyses and for capturing continuous phenotype-dependent molecular interaction dynamics [38, 39]. We aimed to systematically identify key molecular interactions within leukemia-related pathways that influence therapeutic drug response. To achieve this, we used the publicly available Genomics of Drug Sensitivity in Cancer (GDSC) dataset, published by the Cancer Genome Project (https://www.cancerrxgene.org/). The GDSC dataset provides gene expression profiles as well as metrics of anti-cancer drug sensitivity, including half-maximal inhibitory concentration (IC50) values and their corresponding Z-scores. The GDSC dataset including expression levels of genes (i.e. Cell_line_RMA_proc_basalExp.txt) and the drug response dataset (i.e. GDSC1_fitted_dose_response_27Oct23.xlsx) was downloaded in January 2025.

We focused on two major types of leukemia, AML and CML. Additionally, we considered MDS, a hematologic disorder capable of progressing to AML. Genes implicated in the pathways of AML, CML, and MDS were extracted from the Kyoto Encyclopedia of Genes and Genomes (KEGG) pathway database (https://www.genome.jp/kegg/pathway.html). Furthermore, anti-cancer drugs approved for leukemia-related diseases by the Food and Drug Administration (FDA) (https://www.cancer.gov/about-cancer/treatment/drugs/leukemia) were considered. Table 5 presents the number of genes associated with leukemia-related pathways and the 11 approved drugs (O) for AML, MDS, and CML; only drugs with available response data in the GDSC dataset were considered. The gene set comprised 500 genes, including $q$ pathway-related genes and $500-q$ genes selected based on the highest expression variances. To avoid omission of relevant result Drug sensitivity-specific gene networks were estimated across 20 equally distributed quantiles of the Z-score of IC50 values using gene expression data from 100 randomly selected cell lines. Cell lines were selected using simple random sampling without disease-subtype stratification or additional sampling constraints beyond the preprocessing and quality-control procedures applied to the original dataset. The selection of 100 cell lines was informed by previous studies employing related methodological frameworks. Specifically, the kernel-based NetworkProfiler framework, which performs state-dependent gene network inference using a varying-coefficient modeling strategy, was demonstrated using approximately 60 cell lines [14]. The LIONESS framework, a representative method for sample-specific regulatory network inference, was evaluated using 153 RNA-seq samples after quality control [9]. Although not developed for gene network inference, a functional varying coefficient modeling study employing kernel-smoothing estimation evaluated its methodology using 50 and 200 subjects [40], providing a relevant reference because CellGNEA also relies on a varying-coefficient modeling framework for cell-line-specific network estimation. Furthermore, while not itself a kernel-based approach, a recent benchmark study of sample-specific network inference methods in precision oncology applied network reconstruction to cancer-specific datasets containing approximately 50–150 samples, depending on cancer type [34]. Because CellGNEA integrates cell-line-specific network inference with varying-coefficient modeling, these studies provide relevant methodological references for sample-size selection. Collectively, previous studies have commonly employed datasets containing approximately 50–150 samples or cell lines, indicating that the use of 100 cell lines falls within the range typically adopted in related methodological applications. Therefore, we selected 100 cell lines for network estimation as a practical sample size supported by previous studies while maintaining computational feasibility for repeated network reconstruction across multiple IC50 quantiles. Although larger sample sizes may further improve estimation stability, they also increase the computational burden associated with repeated cell-line-specific network estimation. The proposed framework is readily applicable to larger sample sizes when computational resources permit. For example, when inferring the cytarabine-sensitivity-specific gene network, 20 such networks were estimated for the 500 genes, each corresponding to a quartile of the Z-scored IC50 values for cytarabine. If the molecular interactions among AML pathway genes were linked to cytarabine responses, the resulting gene networks were anticipated to display unique characteristics that vary with cytarabine response. More generally, gene network estimation was conducted by leveraging all available cell lines (i.e. 1018 cell lines) with gene expression and drug response data within the GDSC framework.

**Table 5:** Number of genes involved in leukemia-related diseases in the KEGG database and approved anticancer drugs by the U.S. Food and Drug Administration (FDA). “O” indicates that the drug is approved by the U.S. Food and Drug Administration (FDA) for the corresponding disease

	AML	MDS	CML
Entry	hsa05221	H01481	hsa05220
		H02410	
No. Genes	68	22	77
Bosutinib	–	–	O
Cytarabine	O	O	O
Dasatinib	–	O	O
Doxorubicin	O	O	–
Fedratinib	–	O	–
Imatinib	–	O	O
Midostaurin	O	–	–
Nilotinib	–	O	O
Ponatinib	–	–	O
Quizartinib	O	–	–
Ruxolitinib	–	O	–

The proposed CellGNEA was applied to identify leukemia gene networks relevant to drug sensitivities. To investigate potential novel therapeutic agents for leukemia-related diseases beyond existing approved treatments, GNEA was performed for AML, MDS, and CML pathways using all 11 drugs, rather than restricting the analysis to those approved specifically for each disease. Table 6 presents the enrichment analysis results for anti-cancer drug-sensitivity-specific gene networks related to leukemia pathways (FDR–q values).

**Table 6:** Results (FDR q-values) of gene network enrichment analysis of leukemia-related pathways with drug sensitivities.

Drugs	AML	MDS	CML
Bosutinib	4.40e-1	3.00e-3	1.26e-1*
Cytarabine	2.76e-1*	2.97e-1	2.97e-1
Dasatinib	7.20e-2	1.13e-1	9.90e-2
Doxorubicin	1.00e-3*	4.60e-2*	4.60e-2*
Fedratinib	9.60e-2	9.60e-2	3.07e-1
Imatinib	1.00e-3*	1.00e-3*	2.00e-3*
Midostaurin	1.54e-1	4.91e-1	1.54e-1
Nilotinib	1.54e-1*	9.90e-2*	3.00e-3*
Ponatinib	2.70e-1*	1.50e-2*	3.90e-2
Quizartinib	1.00e-3*	1.00e-3*	1.00e-3*
Ruxolitinib	1.00e-3*	1.00e-3*	1.00e-3*

Drugs marked with (*) indicate associations that are consistently significant (FDR–q-values<0.01) in both the original dataset and the independent CCLE-based analysis

As shown in Table 6, doxorubicin, imatinib, quizartinib, and ruxolitinib were identified as being significantly associated with the molecular interplays involved in the AML pathway; bosutinib, imatinib, quizartinib, and ruxolitinib were associated with the MDS pathway; and imatinib, nilotinib, quizartinib, and ruxolitinib were associated with the CML pathway, respectively. Furthermore, several drugs demonstrated significant associations between their responses and gene networks implicated in leukemia-related pathways, despite not being approved for the treatment of this disease. These findings suggest that the identified drugs may serve as novel therapeutic options for leukemia. Of particular interest, imatinib, quizartinib and ruxolitinib exhibited significant associations with gene networks across all leukemia-related pathways (AML, MDS, and CML), highlighting its broad relevance to leukemia-associated molecular interplays.

- Imatinib
  Imatinib is the first clinically used signal transduction inhibitor that targets BCR–ABL in CML by directly inhibiting its constitutive tyrosine kinase activity, thereby blocking proliferative signaling and inducing apoptosis of leukemic cells [41]. Numerous studies have shown that imatinib treatment can achieve complete hematological remission in patients with AML, myeloid sarcoma, and B- and T-cell leukemia/lymphoma associated with the FIP1L1–PDGFRA rearrangement [42].
- Quizartinib
  Quizartinib is a selective FLT3 inhibitor approved for the treatment of AML [43]. Although uniform targeting of the same molecular pathway may be counterproductive [44], Quizartinib has shown encouraging safety and activity in FLT3-mutated MDS and MDS/MPN [45], and has been evaluated mainly in adult populations with limited pediatric and MDS patient data [46].
- Ruxolitinib
  Ruxolitinib is an inhibitor of the Janus kinase (JAK) family of protein tyrosine kinases. Naqvi *et al*. [47] demonstrated its potential utility in the treatment of leukemia, suggesting that ruxolitinib exhibits therapeutic activity in patients with leukemia. Consistent with these findings, accumulating evidence further supports a therapeutic role for ruxolitinib in hematological malignancies [47].

**Figure 4:**
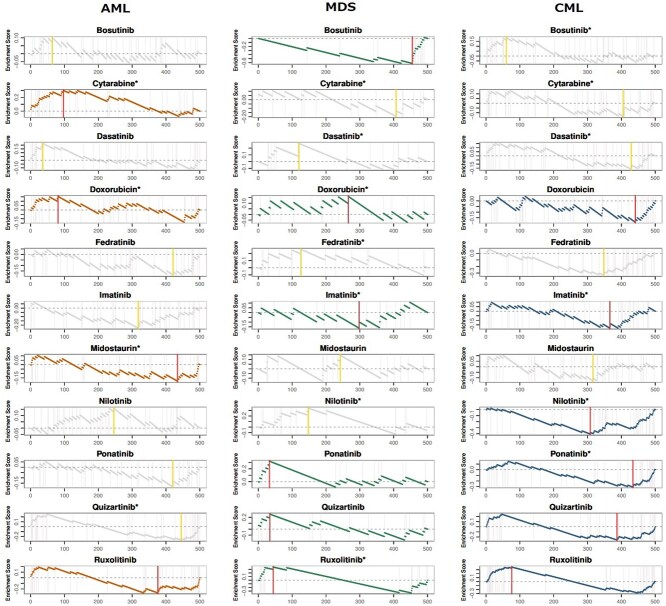
Enrichment score profiles of leukemia-related pathways (AML, MDS, and CML) across IC50-associated ranked gene lists of leukemia drugs. (*) Drugs approved by the FDA for AML, MDS, or CML. The x-axis represents ranked genes ordered according to gene-level statistics associated with IC50 values, and the y-axis represents enrichment scores. The dotted horizontal line indicates zero enrichment score. Red and yellow vertical lines denote the positions of the maximum deviations from zero, corresponding to significant and non-significant enrichment scores, respectively. Orange, green, and blue lines represent statistically significant enrichment results for the AML, MDS, and CML pathways, respectively, whereas gray lines represent non-significant enrichment results. Colors are used solely to indicate pathway type and significance status and do not represent quantitative enrichment magnitude or statistical significance levels.

Figure 4 presents the behaviors of the enrichment score (i.e. $eSC$) for drug sensitivity-specific gene networks. Genes involved in leukemia-related pathways were overrepresented at the bottom of the gene-ranking list derived from the imatinib sensitivity-specific gene networks. In contrast, these genes were overrepresented at the top of the gene-ranking lists derived from gene networks associated with quizartinib and ruxolitinib.

**Figure 5:**
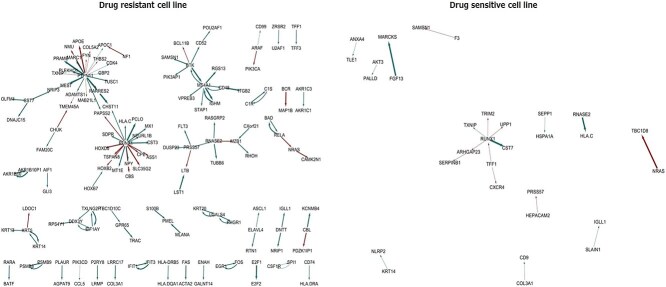
Differential gene regulatory networks across drug-response spectrums. Common gene networks involved in AML, MDS, and CML pathways are compared between drugs (imatinib, quizartinib, and ruxolitinib)-resistant (left) and -sensitive (right) cell lines. (a) Edge colors represent the mode of regulation: green for positive (activating) and red for negative (inhibitory) effects. The thickness of each edge is proportional to the absolute magnitude of the regulatory weight, with thicker lines indicating higher interaction strength. Regulatory direction is illustrated schematically as $\Box \rightarrow \triangle$, where $\Box$ denotes regulator genes (source) and $\triangle$ denotes target genes (sink). These symbols are used only to indicate regulatory directionality and do not correspond to displayed node shapes in the network.

To uncover common molecular architectures of AML, MDS, and CML relevant to the therapeutic response to leukemia drugs, we inferred shared molecular interplays in leukemia-related pathways in the most imatinib, quizartinib and ruxolitinib -sensitive and resistant cell lines. That is, from the gene networks of AML, MDS, and CML in the most sensitive (resistant) cell lines of imatinib, quizartinib and ruxolitinib, we extracted edges that appeared in >30% of the networks (i.e. at least three networks) and computed the median of their edge weights. Figure 5 shows the common gene networks across AML, MDS, and CML pathways for the most imatinib, quizartinib, and ruxolitinib -sensitive and resistant cell lines. To effectively visualize the complex gene networks, we focused on edges corresponding to the top 1% of absolute edge weights.

As shown in Fig. 5, the drugs resistant cell line exhibited considerably more active molecular interplays (i.e. a larger number of edges) than the sensitive cell line. RUNX1 was identified as a hub gene in the leukemia gene networks of both drugs -resistant and -sensitive cell lines; however, their activities were weaker in the sensitive cell lines than in the resistant cell lines. PTPN11 was also identified as a main hub gene in the leukemia pathway gene network for drugs resistant cell line, but its activity disappeared in the network for the sensitive cell line. Similar behavior was observed for BTK and MS4A1. Collectively, these findings suggest that genes that interact exclusively in the resistant cell line may serve as resistance-specific markers associated with leukemia-related pathways.

**Figure 6:**
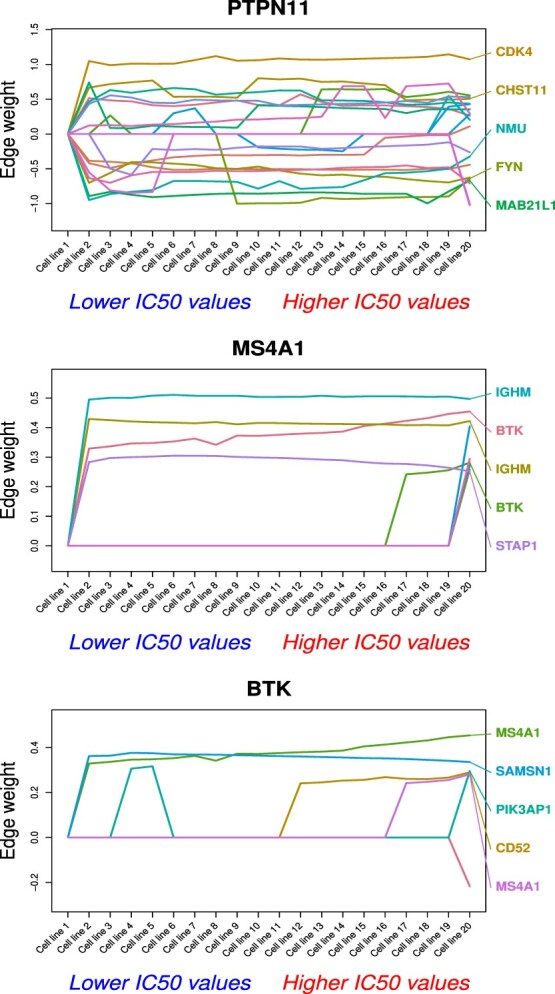
Edge weight trajectories of the marker genes (PTPN11, MS4A1, and BTK) across cell lines ordered according to increasing IC50 values for imatinib, quizartinib, and ruxolitinib. The x-axis represents cell lines continuously ordered along the IC50 spectrum from lower to higher IC50 values, and the y-axis represents edge weights. To improve visualization clarity, only representative interaction partners with the largest absolute edge weights are labeled in the figure. The complete edge weight trajectories across all interaction partners and cell lines are provided in Supplementary Table S2.

To illustrate continuous changes in edge weights across the IC50 spectrum, we computed the median edge weights across 20 cell lines ordered according to increasing IC50 values. The median values were obtained using a procedure similar to that described in Fig. 5. Figure 6 shows the evolution of edge weights for the identified marker genes (i.e. PTPN11, MS4A1, and BTK) across the continuous IC50 spectrum for imatinib, quizartinib, and ruxolitinib. To improve readability, only representative interaction partners with the largest absolute edge weights are labeled in the main figure, whereas the complete edge weight trajectories are provided in Supplementary Table S2.

As shown in Fig. 6, the edge weights exhibit gradual and heterogeneous changes across the IC50 spectrum, rather than abrupt shifts between two discrete states. Notably, the most pronounced changes in edge weights tend to occur at the extremes of the IC50 spectrum, corresponding to lower and higher IC50 regions, whereas transitions in intermediate regions are comparatively more gradual. This suggests that major network rewiring events are concentrated near the boundaries of the IC50 continuum, followed by more subtle adjustments along the spectrum. For PTPN11, multiple edges display varying patterns, with some interactions remaining relatively stable while others fluctuate or gradually increase toward higher IC50 regions, suggesting dynamic rewiring of signaling interactions across the drug-response continuum. For MS4A1, several edges show progressively increasing edge weights along the IC50 spectrum, indicating gradual strengthening of specific interactions associated with drug-response mechanisms. Similarly, BTK exhibits distinct edge-specific trends, where certain interactions gradually intensify toward higher IC50 regions, whereas others emerge only in later regions of the IC50 continuum. These observations suggest that drug response is associated with both continuous modulation and selective activation of gene–gene interactions. Overall, these results demonstrate that drug response is characterized by continuous and gene-specific network rewiring patterns, highlighting the advantage of our framework in capturing dynamic molecular changes across the full IC50 spectrum rather than relying on a dichotomized sensitive-resistant comparison.

**Figure 7:**
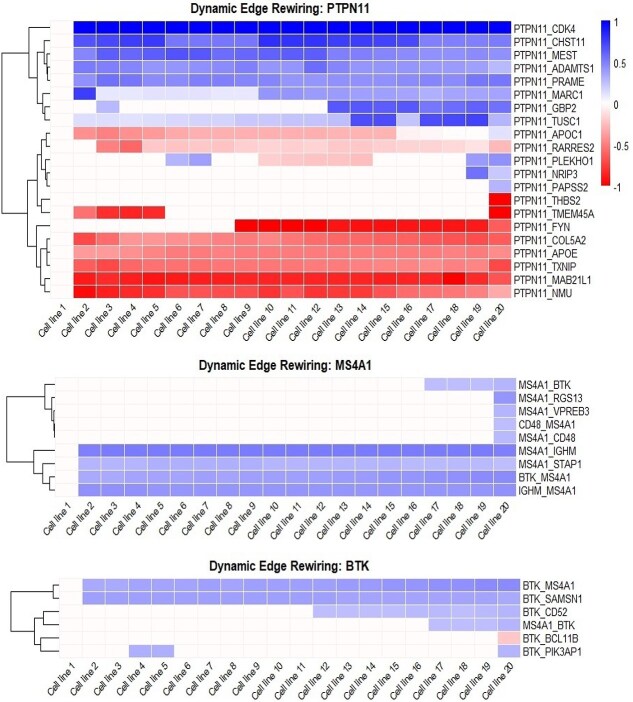
Dynamic edge rewiring and differential network topology linked to resistance evolution. A transition from “Node Scoring” to “Network Mechanism” illustrates specific rewiring events of top-ranked genes associated with increasing drug resistance. The heatmaps display the evolution of edge weights for interaction partners of PTPN11 (top), MS4A1 (middle), and BTK (bottom) across cell-line states ordered from the most sensitive (Cell line 1) to the most resistant (Cell line 20). Because CellGNEA is based on directed cell-line-specific gene networks, edge labels are represented in the form “Regulator${\_}$Target,” where the left and right gene names denote the regulator and target genes, respectively. Consequently, labels such as “MS4A1${\_}$BTK” and “BTK${\_}$MS4A1” represent distinct directed regulatory relationships rather than the same undirected interaction. Interaction partners were hierarchically clustered within each panel according to similarity in dynamic edge rewiring patterns across cell-line states. Therefore, edge ordering and displayed interaction-partner labels reflect clustering-derived network topology and are intended to highlight groups of directed interactions exhibiting similar resistance-associated rewiring behaviors.

To further substantiate the mechanistic transition from static node-level importance to dynamic network-level rewiring, we performed a differential topology analysis focusing on the identified resistance markers (i.e. PTPN11, MS4A1, and BTK). Figure 7 shows the evolution of edge weights for these marker genes along the continuum from drug-sensitive to drug-resistant cell lines. Rather than interpreting these markers as highly ranked individual genes, we examined their local subnetworks to identify edge-specific rewiring events between sensitive and resistant states.

For PTPN11, the analysis reveals a clear reorganization of its interaction landscape. In sensitive states, PTPN11 shows limited associations with key regulators, whereas in resistant cells it gains strong interactions with CDK4 and CHST11, suggesting increased coupling to cell-cycle progression and extracellular matrix remodeling. Additionally, interactions with NRIP3, PAPSS2, THBS2, and FYN emerge specifically in resistant conditions, indicating recruitment of signaling partners linked to survival and microenvironmental adaptation. These edges are weakened or absent in sensitive states, highlighting a loss of these connections upon drug susceptibility and a shift toward a resistance-associated signaling role. MS4A1 exhibits a more selective activation pattern. It remains largely quiescent in sensitive states but, in resistant cells, gains interactions with BTK, RGS13, VPREB3, and CD48. Notably, the MS4A1–BTK connection suggests activation of B-cell receptor-related signaling, while other partners indicate enhanced immune-related communication. The absence of these edges in sensitive states supports a resistance-specific recruitment mechanism. BTK demonstrates a progressive, step-wise rewiring trajectory. Early interactions with SAMSN1 and MS4A1 emerge and persist, followed by strengthened connectivity with CD52 at intermediate stages, and late-stage interactions with BCL11B and PIK3AP1 in highly resistant cells. This sequential gain of edges supports a model of cumulative network reorganization during resistance acquisition. Collectively, these results identify specific gained and lost edges for key resistance markers, demonstrating that drug resistance is driven by dynamic network rewiring rather than static gene-level changes. This edge-centric perspective provides mechanistic insights that cannot be captured by conventional node-based approaches.

**Figure 8:**
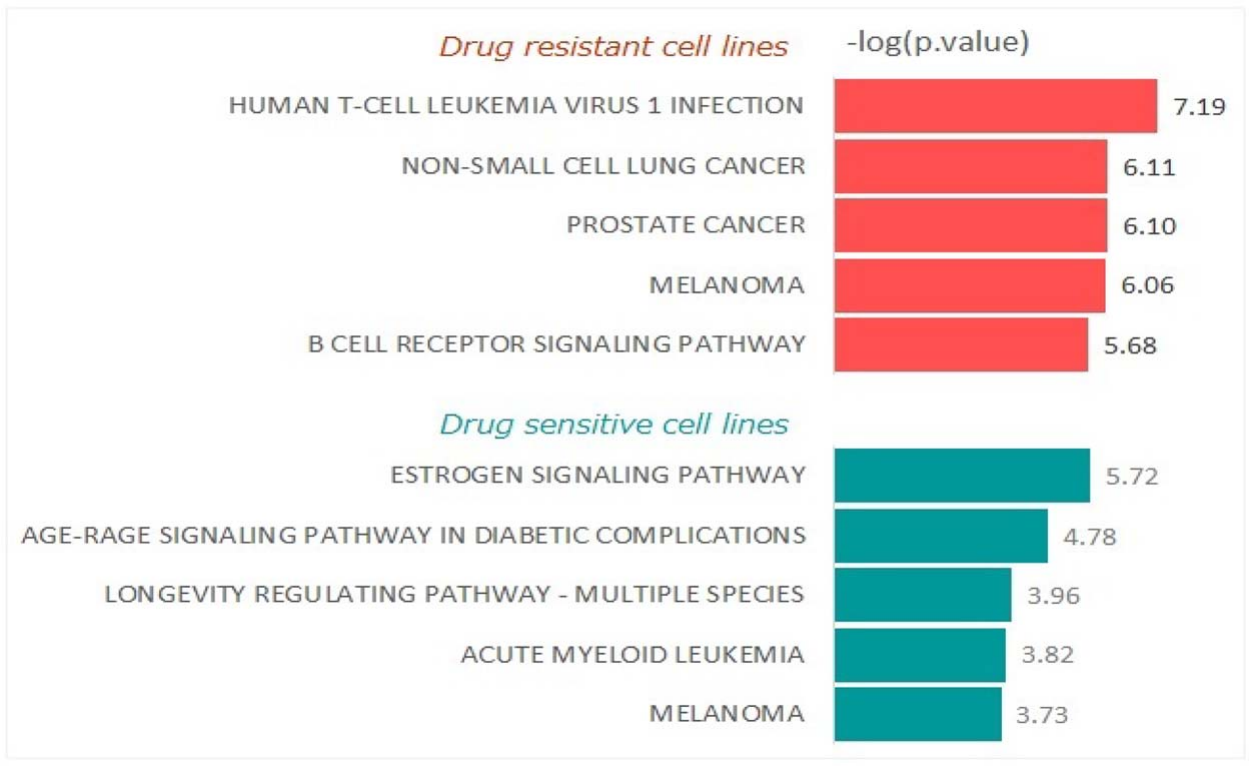
Top five significantly enriched KEGG pathways identified using Enrichr in leukemia drug-sensitive and drug-resistant cell lines, highlighting common molecular interplays across AML, MDS, and CML. Pathways are ranked by $-{\mathrm{log}}10(P$-value).

To investigate the biological functions of common molecular interplays for AML, MDS, and CML in leukemia drug-sensitive and drug-resistant cell lines, KEGG pathway enrichment analysis was performed using Enrichr, a regularly updated bioinformatics tool [48]. We selected the five most significantly enriched pathways ranked by $-{\text{ log}}_{10}(P\mathrm{-value})$ for concise visualization in Fig. 8 [49, 50]. To ensure that all enrichment results are available to readers, the complete, the complete enrichment results are provided in Supplementary Table S3. The gene networks in drug-resistant cell lines exhibited multiple enriched pathway terms; therefore, only the five most significant pathways are presented. “MELANOMA” was identified as a common enriched pathway in both drug-sensitive and drug-resistant cell lines, suggesting shared molecular signaling components across resistance states. In drug-resistant cell lines, enrichment was predominantly observed in cancer- and leukemia-related pathways, including “Human T-cell leukemia virus 1 infection,” “Non-small cell lung cancer,” and “Prostate cancer,” together with immune-related signaling such as the “B cell receptor signaling pathway.” Notably, although the “Human T-cell leukemia virus 1 infection” pathway is virus-associated by annotation, enrichment of this pathway does not imply viral infection itself, but rather reflects dysregulation of shared signaling mechanisms involved in leukemic progression and therapeutic resistance, including oncogenic and immune-associated molecular processes. Collectively, these findings suggest that drug-resistant cell lines are characterized by activation of disease-related and resistance-associated network mechanisms. In contrast, drug-sensitive cell lines showed enrichment predominantly in pathways associated with cellular signaling and systemic regulation, including the “Estrogen signaling pathway,” “AGE–RAGE signaling pathway in diabetic complications,” and “Longevity regulating pathway,” suggesting relatively broader regulatory signaling processes rather than dominant disease-specific network rewiring.

Collectively, our findings and the existing literature indicate that therapeutic targeting and suppression of the interplays among the identified resistance-specific markers (PTPN11, MS4A1, and BTK) and the related pathways may offer important mechanistic insights for leukemia treatment.

To strengthen biological relevance, we performed an additional validation using independent gene expression data from the CCLE. After matching CCLE expression profiles with GDSC drug sensitivity data, we conducted pathway analysis for leukemia-specific gene networks. Drug–pathway associations that remained significant ($P$-value <.01) in both analyses were considered reproducible and are marked with (*) in Table 6. This cross-dataset validation indicates that the identified associations are not dataset-specific but reflect robust and biologically meaningful network-level signals. Table 6 summarizes the results of the gene network enrichment analysis for leukemia-related pathways in relation to drug sensitivities, with validated associations indicated by (*). Several drug–pathway associations remain consistently significant across both the original dataset and the independent CCLE-based analysis, demonstrating strong cross-dataset reproducibility. Notably, clinically relevant drugs, such as imatinib, quizartinib, and ruxolitinib exhibit consistent significance across multiple leukemia subtypes (AML, MDS, and CML), further supporting the biological relevance of the identified associations. In addition, other drugs (e.g. doxorubicin and nilotinib) also show reproducible signals in specific disease contexts, suggesting that the proposed method can capture both well-established and context-specific regulatory mechanisms. Although results from two independent datasets are not expected to be identical, the presence of consistently validated associations across both datasets indicates that the identified signals are not driven by dataset-specific artifacts, but instead reflect stable network-level relationships between drug sensitivity and leukemia-related pathways. These findings provide further evidence that the proposed CellGNEA framework can identify biologically meaningful and reproducible drug–pathway interactions.

Importantly, beyond confirming previously reported associations, the proposed approach provides additional insights into drug–pathway relationships at the network level. Especially, imatinib, quizartinib, and ruxolitinib are consistently identified as significant across all three leukemia subtypes (AML, MDS, and CML) in both datasets. While these drugs are known to be relevant to specific leukemia conditions, they are not universally approved for all three diseases. This cross-dataset and cross-disease consistency suggests that these drugs may exert broader regulatory effects on shared leukemia-related gene networks than previously recognized. Thus, our results highlight their potential as candidate therapeutic agents across multiple leukemia subtypes. This demonstrates that the proposed CellGNEA framework is capable of uncovering reproducible and non-trivial network-level associations that extend beyond established knowledge.

### Functional pathway analysis of gene networks associated with drug sensitivity in breast cancer

To further evaluate the generalizability of the proposed CellGNEA framework beyond leukemia, we applied our approach to an independent breast cancer dataset. We utilized gene expression data from the Cancer Cell Line Encyclopedia (CCLE, DepMap release 22Q2), obtained from the Dependency Map (DepMap) portal (https://depmap.org/portal/), along with drug sensitivity data from the GDSC database. Consistent with the leukemia analysis, we extracted 148 genes involved in breast cancer-related pathways from the KEGG pathway database. A total of 14 FDA-approved drugs for breast cancer were considered for drug sensitivity-associated gene network inference. The breast cancer gene networks were inferred using the same procedure as in the leukemia analysis, ensuring methodological consistency.

Table 7 presents the results of the functional pathway enrichment analysis for the breast cancer drug sensitivity-associated gene networks. The reported P-values were calculated according to Equation (12) using permutation-based significance testing of the CellGNEA pathway enrichment statistic.

**Table 7:** Results ($P$-values) of gene network enrichment analysis for breast cancer-related pathways associated with drug sensitivities. $P$-values were calculated according to Equation (12) using the CellGNEA significance assessment framework with permutation-based significance evaluation. Values are reported in scientific notation; entries previously shown as 1.00e-3 reflect the minimum resolution of permutation-based $P$-value estimates

Drugs	$P$ -value	Drugs	$P$ -value
Capivasertib	1.90e-2	Methotrexate	1.00e-3
Cyclopamine	9.00e-3	Olaparib	4.00e-3
Docetaxel	5.10e-2	Paclitaxel	2.00e-3
Doxorubicin	1.00e-3	Palbociclib	4.00e-3
Fulvestrant	1.10e-2	Talazoparib	1.75e-1
Gemcitabine	1.00e-3	Tamoxifen	5.20e-2
Lapatinib	1.00e-3	Vinblastine	1.00e-1

Results (*p*-values) of gene network enrichment analysis for breast cancer-related pathways associated with drug sensitivities, with underlined values indicating statistically significant results ($p < 0.01$).

**Figure 9:**
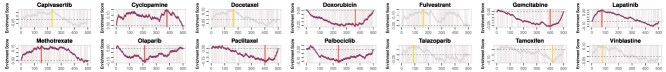
Enrichment score profiles of breast cancer-related pathways across IC50-associated ranked gene lists of breast cancer drugs. The x-axis represents ranked genes ordered according to gene-level statistics associated with IC50 values, and the y-axis represents enrichment scores. The dotted horizontal line indicates zero enrichment score. Red and yellow vertical lines denote the positions of the maximum deviations from zero, corresponding to significant and non-significant enrichment scores, respectively. Purple lines represent statistically significant enrichment results, whereas gray lines represent nonsignificant enrichment results. Colors are used solely to indicate significance status and do not represent quantitative enrichment magnitude or statistical significance levels.

Among the 14 drugs analyzed, half exhibited statistically significant associations ($P$-value <.01) with breast cancer-related pathways, indicating that a substantial proportion of the drugs are functionally linked to the inferred gene networks. The most significant associations were observed for Methotrexate, Doxorubicin, Gemcitabine, and Lapatinib (p = 1.00e-3), suggesting strong enrichment of their corresponding gene networks in breast cancer-related pathways. In addition, several other drugs, including Paclitaxel, Olaparib, and Palbociclib, also demonstrated significant enrichment, further supporting the biological relevance of the identified networks. Figure 9 shows the enrichment score behaviors.

These findings are consistent with previous studies. Doxorubicin resistance in cancer has been associated with autophagy-mediated survival mechanisms, suggesting that targeting autophagy may enhance therapeutic efficacy and help overcome chemoresistance [51]. Xie *et al*. [52] reported that gemcitabine-based combination therapies improve overall survival, progression-free survival, and objective response rates in advanced breast cancer patients, despite increased but manageable hematologic toxicity. Furthermore, lapatinib combined with capecitabine has been shown to significantly reduce the risk of cancer progression in advanced breast cancer without increasing severe adverse effects [53].

We further extracted common molecular interplays from the drug sensitivity-specific gene networks for each of the 14 drugs. Consistent with the leukemia drug sensitivity-specific gene network analysis, we focused on the top 1% of edges with the largest absolute edge weights and retained only those edges that appeared in at least 25% of the networks, i.e. edges present in more than three drug-sensitive and drug-resistant networks. The resulting common gene networks for drug-sensitive and drug-resistant conditions across the significant eight breast cancer drugs are provided in the Supplementary Table S1. In the breast cancer drug-sensitive gene networks, P3R3URF–PIK3R3 (14), FGF4 (6), CSNK1A1L (6), and WNT8A (4) were identified as hub genes, each having more than four edges, where the numbers in parentheses indicate the number of edges. In contrast, in drug-resistant cell lines, CSNK1A1L (18), FGF6 (5), P3R3URF–PIK3R3 (4), and FGF4 (4) were identified as hub genes. Unlike the leukemia drug-associated gene networks, the breast cancer drug-sensitive and drug-resistant gene networks exhibit similar topological characteristics in terms of molecular interplays, particularly hubness. Specifically, P3R3URF–PIK3R3, CSNK1A1L, and members of the FGF gene family appear to play central roles in both drug-sensitive and drug-resistant conditions, suggesting that core regulatory structures are largely preserved across different drug response states in breast cancer.

Our results suggest that targeting and regulating the common hub genes, including P3R3URF–PIK3R3, CSNK1A1L, and members of the FGF gene family, may play a crucial role in breast cancer treatment.

Figure 9 illustrates the enrichment score behaviors for drug sensitivity-associated gene networks. Consistent with the enrichment analysis results, several drugs, including Methotrexate, Doxorubicin, Gemcitabine, and Lapatinib, exhibit pronounced positive deviations from zero, indicating significant enrichment across IC50 values. In contrast, drugs such as Talazoparib, Tamoxifen, and Vinblastine display relatively weak or nonsignificant enrichment patterns, as reflected by smaller deviations from zero. The enrichment profiles of significantly associated drugs show distinct peak patterns, suggesting strong and consistent associations between gene network activity and drug sensitivity.

Taken together, these results demonstrate that the proposed framework successfully captures biologically meaningful drug–pathway relationships in an independent breast cancer dataset, thereby supporting its generalizability beyond leukemia.

## Discussion

In this study, we have proposed CellGNEA, a novel approach tailored for GNEA with continuous phenotypes to identify functionally relevant pathways and molecular interactions in a cell line-specific manner. Within this framework, we successfully estimated cell line-specific gene networks and characterized molecular interactions using various network-driven metrics at the individual cell line level. Subsequently, we assessed the association between these molecular dynamics and continuous phenotypic values. This approach evaluates the significance of these associations using a gene-permutation framework rather than traditional phenotype-label permutation. This strategy addresses a fundamental limitation of existing enrichment analyses, such as GSEA, in which the null distribution is generated by permuting phenotypes, while the test statistic assesses the relative association strength of a gene set, leading to inconsistency between the null model and the tested hypothesis.

The effectiveness of our platform was confirmed using Monte Carlo simulations. By applying CellGNEA to leukemia-related datasets, we systematically uncovered key molecular interactions within leukemia-related pathways linked to therapeutic drug response. Our analysis demonstrated that the response to imatinib, quizartinib, and ruxolitinib are associated with molecular interplays involved in AML, MDS, and CML pathways, and revealed distinct gene regulatory structures between leukemia drugs -sensitive and -resistant cell lines. Furthermore, CellGNEA successfully identified anti-cancer drugs significantly associated with leukemia-related molecular interplays and highlighted resistance-specific markers, such as PTPN11, MS4A1, and BTK, whose roles in leukemia progression and drug resistance are supported by existing literature. The resistance-specific markers identified in this study, including PTPN11, MS4A1, and BTK, have been previously reported in the context of leukemia and drug resistance, supporting the biological relevance of our findings.

- PTPN11
  PTPN11 was identified as a gene whose mutation confers resistance to several newer targeted therapies [54]. Somatic PTPN11 mutations have also been identified in patients with juvenile myelomonocytic leukemia, myelodysplastic syndrome, childhood AML, and acute lymphoblastic leukemia [55].
- MS4A1
  The tumor-suppressive function of MS4A1 has been established and validated in the context of AML progression, with its efficacy associated with immune cell infiltration [56]. Moreover, rituximab resistance is primarily linked to downregulated CD20 expression and MS4A1 (CD20) gene mutations in *de novo* tumors and relapsed/refractory (R/R) disease [57].
- BTK
  Pillinger *et al*. [58] demonstrated that pharmacological inhibition and genetic knockdown of BTK effectively suppress AML blast proliferation, adhesion to bone marrow stromal cells, and cell migration. In CML, BTK is an important marker of imatinib resistance; its expression enhances drug sensitivity, whereas its absence contributes to increased resistance [59]. Consistently, Wang *et al*. [60] further reported that BTK-A overexpression in CML and acute lymphoblastic leukemia is associated with imatinib resistance.

These findings suggest that focusing on modulating interaction networks, rather than targeting individual genes, may yield critical mechanistic insights into leukemia treatment and resistance.

The network metrics used in this study, i.e. clustering coefficient, PageRank, and regulatory effect, are designed to capture complementary aspects of gene function within inferred networks rather than to directly represent experimentally measured regulatory mechanisms [14, 19, 20]. The clustering coefficient quantifies the tendency of neighboring nodes to form locally interconnected structures and has been widely used to characterize modular organization in biological networks [61, 62]. PageRank estimates node importance by recursively incorporating the influence of connected nodes, thereby capturing global centrality and network influence [63, 64]. The regulatory effect integrates gene expression and interaction strength to provide a functional proxy for regulatory relationships within gene networks, motivated by approaches that infer regulatory dependencies from expression-derived network structures [65]. We acknowledge that these measures are indirect and are not derived from direct experimental observations, which may limit biological interpretability. Future work may incorporate experimentally validated regulatory information to further strengthen the biological relevance of the framework.

In summary, CellGNEA provides a powerful framework for elucidating pathway-level mechanisms underlying cell-line biological characteristics, ensuring broad applicability across systems biology and precision medicine.

An important consideration of the proposed framework is that the performance of CellGNEA may depend on the quality and characteristics of the underlying gene network estimation procedure. Our strategy adopted a kernel-based $L_{1}$-type regularization framework for estimating cell-line-specific gene networks. However, as shown in Tables 1 and 4, the enrichment analysis results varied depending on the network inference methodology used. Specifically, the developed CellGNEA with kernel-based $L_{1}$-type regularization approach generally produced improved TPR, ACC, and F1-score values compared with the LIONESS-based approach under several simulation settings, whereas performance patterns differed depending on the proportion of differential edges and the number of genes considered. These results suggest that differences in the inferred network structures can substantially influence downstream pathway enrichment performance. These observations indicate that CellGNEA is sensitive to the properties of the estimated gene regulatory networks. While the proposed framework showed competitive and relatively stable performance across different network estimation settings, the results also suggest that improvements in network inference quality may further enhance the robustness and biological interpretability of CellGNEA. Recent studies have proposed several advanced network inference strategies to improve network estimation accuracy and generalizability across heterogeneous biological datasets. For example, NetREX introduced a context-specific regulatory network reconstruction framework that incorporates prior biological regulatory information together with gene expression data to model network rewiring events [66]. Castro *et al*. [67] proposed a multi-study regulatory network inference framework that leverages shared regulatory patterns across multiple datasets to improve the robustness and accuracy of gene regulatory network estimation. In addition, MetaSEM introduced a meta-learning-based framework for gene regulatory network inference from single-cell RNA sequencing data, demonstrating improved adaptability under high-dimensional and heterogeneous biological settings [68]. Integrating such approaches into the CellGNEA framework may provide more reliable sample-specific network estimation and improve pathway-level enrichment analyses under complex continuous phenotypic settings. Therefore, incorporating prior knowledge-guided, transfer-learning-based, or meta-learning-based network inference frameworks represents an important direction for future research.

### Limitations

Despite the promising performance of CellGNEA, several limitations should be noted. First, the network metrics used in this study, including clustering coefficient, PageRank, and regulatory effect, are designed to capture complementary aspects of gene function within inferred networks rather than to directly represent experimentally validated regulatory mechanisms. While these measures provide useful topological and functional proxies, they are indirect and may limit biological interpretability. Second, the performance of CellGNEA is highly dependent on the accuracy of the inferred cell line-specific gene networks. Since the enrichment analysis is conducted based on these networks, errors, or instability in network inference may substantially affect downstream results and lead to variability in the detected pathway enrichment patterns. Future work will focus on integrating experimentally validated regulatory information and improving the robustness of network inference to enhance the reliability and biological relevance of the framework.

Another limitation of the real-data application is that drug sensitivity-specific gene networks were estimated using 100 randomly selected cell lines. Although this choice was based on previous studies employing related network inference and sample-specific modeling frameworks, the optimal sample size for cell line-specific network estimation remains unclear. Because the number of cell lines may influence network stability, edge-weight estimation, and downstream enrichment results, future studies should systematically investigate the impact of sample size on CellGNEA analyses and establish practical guidelines for sample-size selection in real-world applications.

**Key points**

- CellGNEA was developed to identify pathway-level molecular interactions associated with continuous biological traits.
- CellGNEA infers gene regulatory networks at the individual cell line level and characterizes cell line-specific molecular interactions by integrating multiple topological features.
- CellGNEA resolves the conceptual inconsistency of traditional phenotype-permutation approaches by permuting genes rather than phenotype labels.
- Application of CellGNEA to leukemia drug sensitivity data identified key molecular interactions, resistance markers, and therapeutically relevant drugs, such as imatinib, quizartinib, and ruxolitinib.

## Supplementary Material

Supplementary_material_bbag413

## Data Availability

The code and toy data to implement CellGNEA are available in GitHub https://github.com/HeewonGitHub/CellGNEA.
